# ARIA‐EAACI 2025: Person‐Centred, Digitally Enabled and Artificial Intelligence‐Assisted Change Management in Airway Diseases: An OECD Best Practice for Integrated Care for Chronic Diseases

**DOI:** 10.1002/clt2.70192

**Published:** 2026-08-18

**Authors:** Jean Bousquet, Bernardo Sousa‐Pinto, Mohamed H. Shamji, Maria J. Torres, Ludger Klimek, Holger J. Schünemann, Mario Morais‐Almeida, Rafael José Vieira, Alkis Togias, Boleslaw Samolinski, Arunas Valiulis, Siân Williams, Oliver Pfaar, Torsten Zuberbier, Anna Bedbrook, Wienczyslawa Czarlewski, Maryam Ali Al‐Nesf, Rita Amaral, Josep M. Anto, Antonio Bognanni, Luisa Brussino, Alvaro A. Cruz, Violeta Kvedariene, Habib Douagui, Nikolaos G. Papadopoulos, G. Walter Canonica, Ivan Cherrez‐Ojeda, Mark Dykewicz, Bilun Gemicioglu, Mattia Giovannini, Brigita Gradauskiene, Tari Haahtela, Cristina Jacomelli, Tuomas Jartti, Miloš Jeseňák, Piotr Kuna, Désirée E. Larenas‐Linnemann, Amir H. A. Latiff, Bryan Martin, Yousser Mohammad, Kari Nadeau, Elizabete Nunes, Ken Ohta, Martial Ouédraogo, Padukudru A. Mahesh, Isabella Pali‐Schölll, Ana Margarida Pereira, Frederico S. Regateiro, Nicolas Roche, Mikhail Sofiev, Luis Taborda‐Barata, Charlotte Suppli Ulrik, Sanna K. Toppila‐Salmi, Marylin Valentin Rostan, Leticia de las Vecillas, Maria Teresa Ventura, Giovanni Viegi, De Yun Wang, He Zhang, Luo Zhang, Giorgio Ciprandi, Juan Carlos Ivancevich, Nikolai Khaltaev, Olga Lourenço, Lucas Leemann, Marine Savouré, Juan Jose Yepes‐Nuñez, Arzu Yorgancioglu, Baharudin Abdullah, Mona Al‐Ahmad, Julijana Asllani, Karl‐C. Bergmann, Jonathan A. Bernstein, Michael S. Blaiss, Fulvio Braido, Pedro Carreiro‐Martins, Lorenzo Cecchi, Antonio F. M. Giuliano, George Christoff, Ieva Cirule, Jaime Correia‐de‐Sousa, Elisio M. Costa, Biljana Cvetkovski, Stefano Del Giacco, Philippe Devillier, Dejan Dokic, Maia Gotua, Maria Antonieta Guzman, Elham Hossny, Tomohisa Iinuma, Carla Irani, Zhanat Ispayeva, Kaja Julge, Igor Kaidashev, Kazi S. Bennoor, Helga Kraxner, Inger Kull, Marek Kulus, Maciej Kupczyk, Andriy Kurchenko, Xin Luo, Stefania La Grutta, Lan Le Thi Tuyet, Michael Makris, Branislava Milenkovic, Neven Miculinic, Sang Min Lee, Stephen Montefort, André Moreira, Joaquim Mullol, Rachel Nadif, Alla Nakonechna, Hugo E. Neffen, Stefania Nicola, Marek Niedoszytko, Dieudonné Nyembue, Robyn E. O’Hehir, Ismail Ogulur, Yoshitaka Okamoto, Markus Ollert, Heidi Olze, Oscar Palomares, Petr Panzner, Hae‐Sim Park, Vincenzo Patella, Ruby Pawankar, Constantinos Pitsios, Todor A. Popov, Francesca Puggioni, Santiago Quirce, Agné Ramonaité, Marysia Recto, Maria Susana Repka‐Ramirez, Karla Robles‐Velasco, Menachem Rottem, Marianella Salapatas, Joaquin Sastre, Nicola Scichilone, Juan‐Carlos Sisul, Dirceu Solé, Manuel Soto‐Martinez, Milan Sova, Katarina Stevanovic, Pongsakorn Tantilipikorn, Ana Todo‐Bom, Vladyslav Tsaryk, Ioanna Tsiligianni, Marilyn Urrutia‐Pereira, Erkka Valovirta, Tuula Vasankari, Dana Wallace, Margitta Worm, Osman M. Yusuf, Fares Zaitoun, Mihaela Zidarn, ARIA study group

**Affiliations:** ^1^ Institute of Allergology Charité – Universitätsmedizin Berlin Corporate Member of Freie Universität Berlin and Humboldt‐Universität zu Berlin Berlin Germany; ^2^ Fraunhofer Institute for Translational Medicine and Pharmacology ITMP Immunology and Allergology Berlin Germany; ^3^ ARIA (Allergic Rhinitis and its Impact on Asthma) Montpellier France; ^4^ MEDCIDS – Department of Community Medicine, Information and Health Decision Sciences Faculty of Medicine University of Porto Porto Portugal; ^5^ CINTESIS@RISE– Health Research Network Faculty of Medicine University of Porto Porto Portugal; ^6^ National Heart and Lung Institute Imperial College London UK; ^7^ NIHR Imperial Biomedical Research Centre London UK; ^8^ Allergy Unit Regional University Hospital of Málaga Málaga University ARADyAL Málaga Spain; ^9^ Department of Otolaryngology, Head and Neck Surgery Universitätsmedizin Mainz Mainz Germany; ^10^ Center for Rhinology and Allergology Wiesbaden Germany; ^11^ Department of Health Research Methods, Evidence, and Impact McMaster University Hamilton Ontario Canada; ^12^ Department of Medicine McMaster University Hamilton Ontario Canada; ^13^ Department of Biomedical Sciences Humanitas University Milan Italy; ^14^ Clinical Epidemiology and Research Center (CERC) Humanitas University and Humanitas Research Hospital Milan Italy; ^15^ Allergy Center CUF Descobertas Hospital Lisbon Portugal; ^16^ Division of Allergy, Immunology, and Transplantation (DAIT) National Institute of Allergy and Infectious Diseases, NIH Bethesda Maryland USA; ^17^ Department of Prevention of Environmental Hazards, Allergology and Immunology Medical University of Warsaw Warsaw Poland; ^18^ Institute of Clinical Medicine Medical Faculty of Vilnius University Vilnius Lithuania; ^19^ Institute of Health Sciences Medical Faculty of Vilnius University Vilnius Lithuania; ^20^ Clinic of Asthma, Allergy and Chronic Lung Diseases Vilnius Lithuania; ^21^ International Primary Care Respiratory Group IPCRG Edinburgh UK; ^22^ Section of Rhinology and Allergy Department of Otorhinolaryngology, Head and Neck Surgery University Hospital Marburg Philipps‐Universität Marburg Marburg Germany; ^23^ MASK‐air SAS Montpellier France; ^24^ Adult Allergy and Immunology Division Hamad Medical Corporation Doha Qatar; ^25^ Department of Cardiovascular and Respiratory Sciences Porto Health School Polytechnic Institute of Porto Porto Portugal; ^26^ Department of Women's and Children's Health Paediatric Research Uppsala University Uppsala Sweden; ^27^ Universitat Pompeu Fabra (UPF) Barcelona Spain; ^28^ CIBER Epidemiología y Salud Pública (CIBERESP) Barcelona Spain; ^29^ ISGlobal Barcelona Institute for Global Health Barcelona Spain; ^30^ Department of Medicine Evidence in Allergy Group McMaster University Hamilton Ontario Canada; ^31^ Department of Medical Sciences University of Torino Torino Italy; ^32^ Allergy and Clinical Immunology Unit Mauriziano Hospital Torino Italy; ^33^ Fundacao ProAR and (Faculdade de Medicina da) Universidade Federal da Bahia Salvador Bahia Brazil; ^34^ Faculty of Medicine Institute of Clinical Medicine Clinic of Chest Diseases and Allergology Vilnius University Vilnius Lithuania; ^35^ Department of Pathology Faculty of Medicine Institute of Biomedical Sciences Vilnius University Vilnius Lithuania; ^36^ Service de Pneumo‐Allergologie Centre Hospitalo‐Universitaire de Béni‐Messous Algiers Algeria; ^37^ Allergy Department 2nd Pediatric Clinic University of Athens Athens Greece; ^38^ Personalized Medicine, Allergy & Asthma IRCCS Humanitas Research Hospital Rozzano Milan Italy; ^39^ Universidad Espíritu Santo Samborondón Ecuador; ^40^ Respiralab Research Group Guayaquil Ecuador; ^41^ Section of Allergy and Immunology Saint Louis University School of Medicine Saint Louis Missouri USA; ^42^ Department of Pulmonary Diseases Cerrahpaşa Faculty of Medicine Istanbul University‐Cerrahpaşa Istanbul Turkey; ^43^ Department of Pulmonary Diseases Institute of Pulmonology and Tuberculosis Istanbul University‐Cerrahpaşa Istanbul Turkey; ^44^ Allergy Unit Meyer Children's Hospital IRCCS Florence Italy; ^45^ Department of Health Sciences University of Florence Florence Italy; ^46^ Department of Immunology and Allergology Lithuanian University of Health Sciences Kaunas Lithuania; ^47^ Skin and Allergy Hospital Helsinki University Hospital and University of Helsinki Helsinki Finland; ^48^ Associazione Respiriamo Insieme Padova Italy; ^49^ Department of Pediatrics and Adolescent Medicine Turku University Hospital University of Turku Turku Finland; ^50^ Department of Paediatrics and Adolescent Medicine Jessenius Faculty of Medicine Institute of Clinical Immunology and Medical Genetics University Hospital Martin Martin Slovakia; ^51^ Department of Pulmonology and Phthisiology Jessenius Faculty of Medicine Institute of Clinical Immunology and Medical Genetics University Hospital Martin Martin Slovakia; ^52^ Comenius University Bratislava Slovakia; ^53^ University Hospital Martin Martin Slovakia; ^54^ Division of Internal Medicine, Asthma and Allergy Barlicki University Hospital Medical University of Lodz Lodz Poland; ^55^ Center of Excellence in Asthma and Allergy Médica Sur Clinical Foundation and Hospital Mexico City Mexico; ^56^ Allergy & Immunology Centre Pantai Hospital Kuala Lumpur Kuala Lumpur Malaysia; ^57^ Department of Otolaryngology Division of Allergy & Immunology, Medicine and Pediatrics The Ohio State University Columbus Ohio USA; ^58^ National Center for Research in Chronic Respiratory Diseases Collaborating with WHO‐EMRO Tishreen University School of Medicine Latakia Syria; ^59^ Pharmacy Department Al‐Sham Private University Damascus Syria; ^60^ Department of Environmental Health Harvard T.H. Chan School of Public Health Boston Massachusetts USA; ^61^ Department of Medicine Division of Allergy and Inflammation Beth Israel Deaconess Hospital Boston Massachusetts USA; ^62^ Faculty of Medicine Eduardo Mondlane University Maputo Mozambique; ^63^ Japan Antituberculosis Association (JATA) Fukujuji Hospital Tokyo Japan; ^64^ Department of Pulmonology Yalgado Ouédraogo University Hospital Center Ouagadougou Burkina Faso; ^65^ Department of Respiratory Medicine JSS Medical College JSS Academy of Higher Education and Research Mysuru India; ^66^ Departement of Biological Sciences and Pathophysiology University of Veterinary Medicine Vienna Austria; ^67^ Allergy Unit Instituto and Hospital CUF Porto Portugal; ^68^ Allergy and Clinical Immunology Department Hospitais da Universidade de Coimbra Unidade Local de Saúde de Coimbra Coimbra Portugal; ^69^ Faculty of Medicine Center for Innovative Biomedicine and Biotechnology (CIBB) University of Coimbra Coimbra Portugal; ^70^ Faculty of Medicine Institute of Immunology University of Coimbra Coimbra Portugal; ^71^ Faculty of Health Sciences RISE‐Health University of Beira Interior Covilhã Portugal; ^72^ UBIAir—Clinical & Experimental Lung Centre University of Beira Interior Covilhã Portugal; ^73^ Pneumologie AP‐HP Centre Université de Paris Cité Hôpital Cochin Paris France; ^74^ UMR 1016 Institut Cochin Paris France; ^75^ Inserm Equipe d’Epidémiologie Respiratoire Intégrative CESP Villejuif France; ^76^ Finnish Meteorological Institute (FMI) Helsinki Finland; ^77^ Department of Immunoallergology Local de Saúde Cova da Beira Covilhã Portugal; ^78^ Department of Respiratory Medicine Copenhagen University Hospital‐Hvidovre Copenhagen Denmark; ^79^ Institute of Cinical Medicine University of Copenhagen Copenhagen Denmark; ^80^ Department of Otorhinolaryngology University of Eastern Finland Kuopio Finland; ^81^ North Savo Wellbeing Services County Kuopio Finland; ^82^ Department of Allergy Skin and Allergy Hospital Inflammation Center Helsinki University Hospital and University of Helsinki Helsinki Finland; ^83^ Pediatrics, Allergy & Immunology Latín American Society of Allergy, Asthma & Immunology (SLAAi) Montevideo Uruguay; ^84^ Department of Allergy Hospital La Paz Institute for Health Research (IdiPAZ) Madrid Spain; ^85^ University of Bari Medical School Bari Italy; ^86^ Institute of Sciences of Food Production National Research Council (ISPA‐CNR) Bari Italy; ^87^ Pulmonary Environmental Epidemiology Unit CNR Institute of Clinical Physiology Pisa Italy; ^88^ Department of Otolaryngology Yong Loo Lin School of Medicine National University of Singapore Singapore; ^89^ Department of Otolaryngology‐Head and Neck Surgery The Third Affiliated Hospital of Sun Yat‐sen University Guangzhou China; ^90^ Casa di Cura Villa Montallegro Genova Italy; ^91^ Servicio de Alergia e Immunologia Clinica Santa Isabel Buenos Aires Argentina; ^92^ Global NCD Platform Geneva Switzerland; ^93^ RISE‐Health Department of Medical Sciences Faculty of Health Sciences University of Beira Interior Covilhã Portugal; ^94^ Department of Political Science University of Zurich Zurich Switzerland; ^95^ School of Medicine Universidad de los Andes Bogotá DC Colombia; ^96^ Fundación Santa Fe de Bogotá Bogotá DC Colombia; ^97^ Department of Pulmonary Diseases Faculty of Medicine Celal Bayar University Manisa Turkey; ^98^ Department of Otorhinolaryngology, Head and Neck Surgery School of Medical Sciences Universiti Sains Malaysia Kubang Kerian Kelantan Malaysia; ^99^ Microbiology Department College of Medicine Kuwait University Kuwait City Kuwait; ^100^ Department of Internal Medicine University of Medicine Tirana Albania; ^101^ Division of Immunology, Allergy and Rheumatology Department of Medicine University of Cincinnati College of Medicine Cincinnati Ohio USA; ^102^ Department of Pediatrics Medical College of Georgia at Augusta University Augusta Georgia USA; ^103^ Respiratory and Allergy Clinic IRCCS – Policlinico San Martino Genova Italy; ^104^ Department of Internal Medicine (DIMI) University of Genoa Genova Italy; ^105^ CHRC LA‐REAL NOVA Medical School NMS Universidade NOVA de Lisboa Lisboa Portugal; ^106^ Serviço de Imunoalergologia Hospital de Dona Estefânia ULS São José Lisbon Portugal; ^107^ Allergy and Clinical Immunology Unit San Giovanni di Dio Hospital Florence Italy; ^108^ Clinica Medica ‘A. Murri’ Department of Preventive and Regenerative and Ionian Area (DiMePre‐J) University of Bari “Aldo Moro” AOUC Policlinico Hospital Bari Italy; ^109^ Bulgarian Alliance for Clinical and Translational Allergy Sofia Bulgaria; ^110^ Latvian Association of Allergists University Children Hospital Riga Latvia; ^111^ Life and Health Sciences Research Institute (ICVS) School of Medicine University of Minho Braga Portugal; ^112^ CINTESIS@RISE Biochemistry Lab Faculty of Pharmacy and Competence Center on Active and Healthy Ageing University of Porto Porto Portugal; ^113^ Woolcock Institute of Medical Research Sydney New South Wales Australia; ^114^ Department of Medical Sciences and Public Health and Unit of Allergy and Clinical Immunology University Hospital “Duilio Casula” University of Cagliari Cagliari Italy; ^115^ VIM Suresnes, UMR 0892, Pôle des Maladies des Voies Respiratoires Hôpital Foch Université Paris‐Saclay Suresnes France; ^116^ Medical Faculty Skopje University Clinic of Pulmology and Allergy Skopje North Macedonia; ^117^ Center of Allergy and Immunology, and Georgian Academy of Allergy, Asthma, and Clinical Immunology Tbilisi Georgia; ^118^ Immunology and Allergy Division Clinical Hospital University of Chile Santiago Chile; ^119^ Pediatric Allergy Immunology and Rheumatology Unit Children's Hospital Ain Shams University Cairo Egypt; ^120^ Department of Otorhinolaryngology Chiba University Chiba Japan; ^121^ Department of Internal Medicine and Infectious Diseases St Joseph University Hotel Dieu de France Hospital Beirut Lebanon; ^122^ Department of Allergology and Clinical Immunology Kazakhstan Association of Allergology and Clinical Immunology Kazakh National Medical University Almaty Kazakhstan; ^123^ Institute of Clinical Medicine Tartu University Children's Clinic Tartu Estonia; ^124^ Poltava State Medical University Poltava Ukraine; ^125^ Department of Respiratory Medicine National Institute of Diseases of the Chest and Hospital Dhaka Bangladesh; ^126^ Department of Otorhinolaryngology, Head and Neck Surgery Semmelweis University Budapest Hungary; ^127^ Department of Clinical Science and Education Södersjukhuset Karolinska Institutet Stockholm Sweden; ^128^ Sach's Children and Youth Hospital Södersjukhuset Stockholm Sweden; ^129^ Department of Pediatric Respiratory Diseases and Allergology Medical University of Warsaw Warsaw Poland; ^130^ Department of Clinical and Laboratory Immunology Allergology and Medical Genetics Bogomolets National Medical University Kyiv Ukraine; ^131^ Institute of Translational Pharmacology (IFT) – National Research Council (CNR) Palermo Italy; ^132^ Asthma COPD Outpatient Care Unit University Medical Center Ho Chi Minh City Vietnam; ^133^ Allergy Unit “D Kalogeromitros” 2nd Department of Dermatology and Venereology National & Kapodistrian University of Athens “Attikon” University Hospital Athens Greece; ^134^ Faculty of Medicine Clinic for Pulmonary Diseases Clinical Center of Serbia University of Belgrade Serbian Association for Asthma and COPD Belgrade Serbia; ^135^ Croatian Pulmonary Society Zagreb Croatia; ^136^ Division of Respiratory Disease and Allergy Department of Internal Medicine Dankook University College of Medicine Cheonan South Korea; ^137^ Department of Medicine Faculty of Medicine and Surgery University of Malta Msida Malta; ^138^ EPIUnit Institute of Public Health University of Porto Porto Portugal; ^139^ Laboratory for Integrative and Translational Research in Population Health (ITR) Porto Portugal; ^140^ Serviço de Imunoalergologia Centro Hospitalar Universitário São João Porto Portugal; ^141^ Basic and Clinical Immunology Unit Department of Pathology Faculty of Medicine University of Porto Porto Portugal; ^142^ Rhinology Unit & Smell Clinic ENT Department Hospital Clínic Barcelona Spain; ^143^ Clinical & Experimental Respiratory Immunoallergy FRCB‐IDIBAPS CIBERES University of Barcelona Barcelona Spain; ^144^ Université Paris‐Saclay UVSQ Université Paris‐Sud Paris France; ^145^ Imperial College Healthcare NHS Trust London UK; ^146^ University of Liverpool Liverpool UK; ^147^ Center of Allergy, Immunology and Respiratory Diseases Santa Fe Argentina; ^148^ Department of Allergology Medical University of Gdańsk Gdansk Poland; ^149^ ENT Department University Hospital of Kinshasa Kinshasa Democratic Republic of the Congo; ^150^ Allergy, Asthma and Clinical Immunology Alfred Health Department of Immunology School of Translational Medicine Monash University Melbourne Victoria Australia; ^151^ Swiss Institute of Allergy and Asthma Research (SIAF) University of Zurich Davos Switzerland; ^152^ Chiba University Hospital Chiba Japan; ^153^ Chiba Rosai Hospital Chiba Japan; ^154^ Department of Infection and Immunity Luxembourg Institute of Health Esch‐sur‐Alzette Luxembourg; ^155^ Department of Dermatology and Allergy University Medical Center UZ Brussels Vrije Universiteit Brussel (VUB) Jette/Brussels Belgium; ^156^ Department of Otorhinolaryngology Charité ‐ University Medical Center Berlin Berlin Germany; ^157^ Department of Biochemistry and Molecular Biology School of Chemistry Complutense University of Madrid Madrid Spain; ^158^ Department of Immunology and Allergology Faculty of Medicine in Pilsen Charles University Prague Czech Republic; ^159^ Department of Allergy and Clinical Immunology Ajou University School of Medicine Suwon South Korea; ^160^ Division of Allergy and Clinical Immunology Department of Medicine ‘Santa Maria della Speranza’ Hospital Salerno Italy; ^161^ Agency of Health ASL Salerno Italy; ^162^ Postgraduate Programme in Allergy and Clinical Immunology University of Naples Federico II Naples Italy; ^163^ Department of Pediatrics Nippon Medical School Tokyo Japan; ^164^ Medical School University of Cyprus Nicosia Cyprus; ^165^ Clinic of Occupational Diseases University Hospital Sveti Ivan Rilski Sofia Bulgaria; ^166^ Department of Pulmonology and Allergology Klaipeda National Hospital Klaipeda Lithuania; ^167^ Vilnius University Medical Faculty Vilnius Lithuania; ^168^ Division of Adult and Pediatric Allergy and Immunology University of the Philippines – Philippines General Hospital Manila Philippines; ^169^ Department of Allergy Clinics Hospital National University San Lorenzo Paraguay; ^170^ LEADER Research Inc Hamilton Ontario Canada; ^171^ Division of Allergy Asthma and Clinical Immunology Emek Medical Center Afula Israel; ^172^ Rappaport Faculty of Medicine Technion‐Israel Institute of Technology Haifa Israel; ^173^ ARIA, Asthma, Rhinitis, Immunology & Allergy Department Athens Greece; ^174^ Allergy Service Fundacion Jimenez Diaz Autonoma University of Madrid CIBERES‐ISCIII Madrid Spain; ^175^ PROMISE Department University of Palermo Palermo Italy; ^176^ Allergy & Asthma CLINICA SISUL FACAAI SPAAI Asuncion Paraguay; ^177^ Division of Allergy, Clinical Immunology and Rheumatology Department of Pediatrics Federal University of São Paulo São Paulo Brazil; ^178^ Division of Respiratory Medicine Department of Pediatrics Hospital Nacional de Niños Universidad de Costa Rica San Jose Costa Rica; ^179^ Department of Respiratory Medicine and Tuberculosis University Hospital Brno Czech Republic; ^180^ Department of Otolaryngology Faculty of Medicine Siriraj Hospital Center of Research Excellence in Allergy & Immunology Mahidol University Bangkok Thailand; ^181^ Imunoalergologia Centro Hospitalar Universitário de Coimbra Faculty of Medicine University of Coimbra Coimbra Portugal; ^182^ International Primary Care Respiratory Group IPCRG Aberdeen UK; ^183^ Health Planning Unit Department of Social Medicine Faculty of Medicine University of Crete Heraklion Greece; ^184^ Federal University of Pampa Uruguaiana Brazil; ^185^ Department of Lung Diseases and Clinical Immunology University of Turku Turku Finland; ^186^ FiLHA Finnish Lung Health Association Helsinki Finland; ^187^ Department of Clinical Medicine Pulmonary Diseases and Clinical Allergology University of Turku Turku Finland; ^188^ Nova Southeastern University College of Allopathic Medicine Fort Lauderdale Florida USA; ^189^ Division of Allergology and Immunology Department Dermatology, Venerology and Allergology Charité Universitätsmedizin Berlin Berlin Germany; ^190^ The Allergy and Asthma Institute Islamabad Pakistan; ^191^ Lebanese‐American University Clemenceau Medical Center DHCC Dubai UAE; ^192^ University Clinic of Respiratory and Allergic Diseases Golnik Slovenia; ^193^ Faculty of Medicine University of Ljubljana Ljubljana Slovenia

**Keywords:** ARIA, artificial intelligence, asthma, mHealth, rhinitis

## Abstract

Allergic Rhinitis and its Impact on Asthma (ARIA) was, up until 2017, a guideline using the best evidence (Grading of Recommendations, Assessment, Development and Evaluation, GRADE) and developed as a change management strategy. A second change management strategy—in collaboration with the European Academy of Allergy and Clinical Immunology (ARIA‐EAACI)—was developed as a person‐centred, digitally enabled, artificial intelligence‐assisted care (person‐centred care) with strong political involvement. The digital tools of ARIA are mainly based on MASK‐air, an Organisation for Economic Co‐operation and Development (OECD) Best Practice for integrated care for chronic diseases. Artificial intelligence was used, in particular, to approach the patients' views and expectations. The current paper describes the steps to build and achieve a new change management strategy. The future of the Change Management strategy is (i) a collaboration between ARIA and EAACI, (ii) the development of ARIA 2024‐2025 guidelines, (iii) the new ARIA‐MeDALL classification of multimorbid airway diseases and (iv) embedding MASK‐air in a registry for severe allergic diseases. The ultimate goals of the ARIA‐EAACI change management strategy will be (i) the transformation of health and care in rhinitis and asthma multimorbidity and (ii) the development of novel guidelines and policies in a cost‐effective manner, improving shared‐decision‐making.

**Abbreviations**
AD: atopic dermatitis
AHA: Active and Healthy Ageing
AI: artificial intelligence
AIRWAYS ICPs: integrated care pathways for airway diseases
AIT: allergen immunotherapy
AR: allergic rhinitis
ARIA: Allergic Rhinitis and its Impact on Asthma
Aze‐Flu: azelastine‐fluticasone propionate
C: conjunctivitis
CARAT: Control of Allergic Rhinitis and Asthma Test
Catalyse: Climate Action to Advance HeaLthY Societies in Europe
CDSS: clinical decision support system
CM: change management
CM2: second phase of change management
CSMS: combined symptom‐medication score for allergic diseases
DG: Directorate General
DG CONNECT: Directorate General for Communications Networks, Content & Technology
DG Santé: Directorate General for Health and Food Safety
e‐DASTHMA: electronic daily control medication score for asthma
EAACI: European Academy of Allergy and Clinical Immunology
EGEA: Epidemiological study on the Genetics and Environment of Asthma, bronchial hyperresponsiveness and atopy
EIP on AHA: European Innovation Partnership on Active and Healthy Ageing
EQ‐5D: EuroQol
ERS: European Respiratory Society
F: formoterol
GA^2^LEN: Global Allergy and Asthma Excellence Network
GARD: WHO Global Alliance against Chronic Respiratory Diseases
GDPR: General Data Protection Regulation
HCP: healthcare professional
ICP: integrated care pathway
ICS: inhaled corticosteroid
ICT: information and communication technology
IN: intranasal
INAH: intranasal H1‐anti‐histamine
INCS: intranasal corticosteroid
IT: information technology
ITU: International Telecommunication Union
LABA: Long‐acting ß2 agonist
MACVIA: Contre les MAladies Chroniques pour un VIeillissement Actif
MASK‐air: Mobile Airways Sentinel networK
MeDALL: Mechanisms of the Development of Allergy
mHealth: mobile health
OAH: oral H1‐anti‐histamine
OECD: Organisation for Economic Co‐operation and Development
OTC: over‐the‐counter
PCC: Person‐centred care
POLLAR: Impact of air POLLution on Asthma and Rhinitis
PROM: patient‐reported outcome measure
QOL: quality of life
RCT: randomised controlled trial
SABA: short‐acting ß2 agonist
SCUAD: severe chronic upper airway disease
SDM: shared decision making
UCRAID: Ukrainian Citizen and refugee electronic support in Respiratory diseases, Allergy, Immunology and Dermatology
VAS: visual analogue scale
WHO: World Health Organization
WPAI‐AS: Work Productivity and Activity questionnaire

## 1 Introduction

In all societies, the disease burden and the healthcare costs for people with allergic and chronic respiratory diseases are increasing rapidly [1]. There is a need to support the transformation of the healthcare system for integrated care through innovative technology including digital health [2] and artificial intelligence (AI) [3]. However, patient acceptability, safety and equitable access to health care need to be ascertained, and ethical and practical issues should be addressed [4].

The traditional healthcare model is focused on diseases (medicine and natural science) and does not acknowledge patients' resources and abilities to be experts in their own life based on their lived experiences. Improving healthcare safety, quality and coordination, as well as quality of life, are important aims in the care of patients with chronic conditions [5]. Patient‐centred care (PCC) is an approach meeting these aims. It increases patients' involvement in their own health [6], and is essential for a successful healthcare transformation. Digital tools are promoters for PCC practices in chronic care [7]. Digital tools and functionality specifically targeted to objectives and outcomes of the person help to achieve PCC, although, alone, they cannot achieve the ideals of PCC [8].

Since 1999, Allergic Rhinitis and its Impact on Asthma (ARIA) has evolved from the first multi‐morbidity guideline in respiratory diseases using the best current evidence‐based models [9, 10, 11, 12, 13] to integrated care pathways (ICPs) using digital tools in AR and asthma multimorbidity (Mobile Airways Sentinel networK: MASK‐air) [14, 15, 16] as an exemplar for the digital transformation of health and care in chronic diseases [17, 18]. It has followed a change management (CM) strategy using the Kotter's model [19] and a framework was proposed in 2019 for CM using MASK‐air [20].

ARIA in collaboration with EAACI should not be considered only as a guideline but also as a fully developed CM strategy using a digitally enabled (based on MASK‐air), AI‐assisted PCC with strong political involvement. It is an OECD (Organisation for Economic Co‐operation and Development) Best Practice for integrated care for chronic diseases [18]. The ultimate goal to be achieved will be the transformation of health and care in rhinitis and asthma multimorbidity and the development of novel guidelines and policies in order to optimise shared decision‐making.

## 2 MASK‐Air

MASK‐air is a validated mHealth app (Medical Device regulation Class IIa) centred around the patient to improve patient‐centred management of allergic rhinitis (AR) and asthma (A) [21]. The MASK‐air app [22] is freely operational in 35 countries. Over 75,000 users have been registered. It is a Good Practice of DG Santé on digitally enabled, person‐centred care [17]. It is also a Best Practice of OECD for integrated care for chronic diseases [18]. It has been certified by the Polish and Ukrainian Governments (April 2024).

The MASK‐air app includes a daily questionnaire (visual analogue scales (VAS) for global allergy symptoms, nose, eye, asthma, work or education [22] and EQ‐5D [23]), as well as daily medications customised for each country and regularly updated using a scroll list questionnaire. It takes 1–2 minutes each day to fill‐in. Digital daily symptom‐medication scores for allergy (CSMS) [24] or asthma (e‐DASTHMA) [25] are calculated and included in the app. Electronic shared decision‐making is available daily using a very simple tool using General Data Protection Regulation (GDPR) requirements and a QR code. Additive questionnaires (Control of Allergic Rhinitis and Asthma Test: CARAT, a patient‐reported outcome measure (PROM) that assesses the level of control of both asthma and AR using a single tool over a period of 4 weeks [26]; EQ‐5D utilities [23] and the Work Productivity and Activity Impairment Allergic Specific (WPAI‐AS) questionnaire [27]) are included. Pollen counts and air pollution are available daily on a 5 km radius in Europe [28]. MASK‐air can be used in adolescents and the elderly [29]. Maturity level [30] and methodologic validation are presented in Supporting Information S1: Tables S1 and S2.

The strategic research objectives of MASK‐air were followed through WHO‐associated projects, EU grants and projects as well as ARIA‐EAACI (European Academy of Allergy and Clinical Immunology) Task Forces (Supporting Information S1: Table S3).

MASK‐air promotes a PCC personalised medicine with optimal shared‐decision making.

## 3 Digital Biomarkers in Airway Diseases

ARIA and EAACI (European Academy of Allergy and Clinical Immunology) have developed several Task Forces and Position Papers aimed at proposing electronic patient‐reported outcome measures (PROMs) as digital biomarkers [31, 32, 33]. First, control digital biomarkers were defined to make a bridge between clinical practice, randomised controlled trials (RCTs), observational real‐life studies and allergen challenges. Using the MASK‐air app as a model, a daily electronic combined symptom‐medication score for allergic diseases (CSMS) [24] or for asthma (e‐DASTHMA) was proposed [25]. To mimic real‐life, it secondly proposed quality‐of‐life digital biomarkers including daily EQ‐5D visual analogue scales. Finally, patient satisfaction with current treatment was also embedded [34]. A strategy has been proposed to follow patients with AIT [33] or biologics for severe asthma [32].

## 4 Novel Airway Phenotypes: Rhinitis Alone Is Distinct From Rhinitis and Asthma

With new data provided mainly by MeDALL (Mechanisms of the Development of Allergy, EU grant) [35, 36] and MASK‐air [22], the concept of R and A proposed by ARIA [10] needed to be re‐assessed [37]. These data included (i) insights into polysensitisation and multimorbidity, (ii) advances in mHealth for novel phenotype definition, (iii) confirmation in canonical epidemiologic studies, (iv) genomic findings, and (v) treatment approaches. The new data have led to novel concepts on the onset of rhinitis and multimorbidity.

The ‘one‐airway‐one‐disease’ concept does not always hold true, and several phenotypes can be defined. These include an extreme ‘allergic’ (asthma) phenotype combining asthma, rhinitis and conjunctivitis [38, 39, 40, 41]. Rhinitis alone and rhinitis and asthma multimorbidity represent two distinct diseases with the following differences [37]: (i) genomic and transcriptomic background (Toll Like Receptors and IL‐17 for rhinitis alone as a local disease; IL‐33 and IL‐5 for allergic and non‐allergic multimorbidity as a systemic disease) [42, 43], (ii) allergen sensitisation patterns (mono‐ or pauci‐sensitisation vs. polysensitisation) [44, 45, 46], (iii) severity of symptoms and (iv) treatment response [47]. In conclusion, the ARIA‐MeDALL hypothesis [37] proposed that rhinitis alone (local disease) and rhinitis with asthma multimorbidity (systemic disease) should be considered as two distinct diseases, possibly modulated by the microbiome. This may be a model for understanding the epidemics of chronic and auto‐immune diseases.

## 5 Artificial Intelligence

Artificial intelligence plays a crucial role in bridging the gap between clinical research and real‐world patient experiences. It enables the analysis of large datasets from over 75,000 MASK‐air users across 35 countries, revealing patterns in symptom‐medication relationships, treatment adherence, and disease progression that are difficult to detect with traditional methods. Most importantly, AI captures unbiased patient perspectives and preferences that were previously missing from guideline development [48]. This allows for evidence‐based recommendations that genuinely reflect patient needs rather than just clinical assumptions. A substantial part of the technological framework relies on data‐driven approaches that may involve ‘machine learning’. Machine learning is one of the methods of AI that uses algorithms trained on data to produce models that can perform those complex tasks. Machine learning is used to perform defined tasks such as categorising data or making predictions, and is narrower than generative AI.

The technology also integrates real‐world evidence with randomised controlled trial data, supporting the creation of personalised care pathways and early identification of patients who may need intervention [49]. AI helps distinguish between different airway disease phenotypes [50], especially the critical difference between rhinitis alone and rhinitis with asthma multimorbidity, marking a shift towards precision medicine. Although in early stages of implementation, the ability of AI to process daily symptom data, environmental factors, and treatment responses positions it as an essential component of truly digital, person‐centred care. This approach aims to enhance shared decision‐making between patients and healthcare providers.

While AI offers significant potential, its implementation within ARIA‐EAACI 2025 must strictly follow ethical guidelines and safety standards [51]. Protecting patient data privacy and security is essential, requiring compliance with GDPR regulations, along with measures such as MASK‐air's Privacy Impact Assessment and oversight by an external Data Protection Officer, already in place with MASK‐air. AI algorithms must undergo transparent validation across diverse populations to prevent bias and promote equitable access—especially important given ARIA's global scope, including low‐ and middle‐income countries where healthcare disparities are common [52]. There is a risk that AI could inadvertently reinforce existing healthcare inequalities if not carefully monitored and validated across different demographic, cultural, and socioeconomic groups. It is important to maintain clinical oversight to ensure that AI supports, rather than replaces, human judgement and helps preserve the patient‐physician relationship. Patients should be fully informed about how their data are used, with clear consent processes and control over their information. Currently, AI development is at Technology Readiness Level TRL 3 to 4, indicating the need for ongoing testing, validation, and improvement before widespread deployment. Ultimately, AI in health care should aim to enhance, not undermine, key medical ethics principles: autonomy, beneficence, non‐maleficence, and justice.

## 6 ARIA 2017: Change Management in Airway Diseases

Asthma, rhinitis and atopic dermatitis (AD) are interrelated clinical phenotypes that partly overlap in the human interactome. Allergic Rhinitis and its Impact on Asthma (ARIA) is a CM approach of the links between upper‐ and lower‐airway allergic diseases using the 8‐step model of Kotter to assess and implement the impact of rhinitis on asthma multimorbidity and to propose multimorbid guidelines [20]. The ARIA 2017 guideline [13] was used as a model of guideline interpretation [20, 53]. However, it did not include the patient's views which were further added [54].

## 7 ARIA‐EAACI 2025 Achievements for a Novel Change Management

A second CM (CM2) was proposed by ARIA Phase 4 in 2019 to increase self‐management and shared decision‐making in rhinitis and asthma multimorbidity [20]. An innovation of this strategy has been the development and validation of IT evidence‐based tools (MASK‐air) that can inform patient decisions on the basis of a self‐care plan proposed by the healthcare professional. The goal was to propose a digitally enabled PCC. We also embedded AI at a later stage. We have now completed CM2.

### 7.1 Step 1: Create Urgency

Several unmet needs in AR and asthma multimorbidity still exist and there is a need for a more effective healthcare system that includes a true person‐centred, cost‐effective management including digital health and AI. (Recommendations, Assessment, Development and Evaluation)‐based guidelines for physicians are available for AR but they are not person‐centred [13, 54].

### 7.2 Step 2: Form a Powerful Coalition

The ARIA network and the MASK‐air think tank represent over 500 members in over 70 countries. They are very active as a coalition and are regularly requested to be involved in different ARIA or MASK‐air initiatives [22, 30, 32, 37, 54, 55, 56, 57, 58, 59, 60, 61, 62, 63]. The coalition includes people deploying the CM vision with all stakeholders, from citizens to patients, health and social care professionals, media and policy makers. The coalition was a GARD (WHO Global Alliance against Chronic Respiratory Diseases) demonstration project [64]. Finally, the transfer of innovation of ARIA has been carried out by the Reference Sites of the European Innovation Partnership on Active and Healthy Ageing [65]. The collaboration now includes EAACI with all its network.

### 7.3 Step 3: Create a Vision for Change

The vision of the ARIA‐EAACI collaboration is to provide a novel feasible and achievable person‐centred CM strategy for AR and A multimorbidity in order to optimise shared decision‐making with the ultimate goal of improving AR and A control while maintaining quality‐of‐life and reducing costs. The short‐term wins were achieved with a digitally enabled, person‐centred care (Table 1). In this second phase, we attempted to finalise the vision including AI and to obtain a large political approval.

**TABLE 1:** Short‐term wins for the second change management (updated from [22]).

Study name	Ref	Study type	*N* users	*N* days	*N* countries
Baseline characteristics					
Pilot study of mobile phone technology in AR in European countries. The MASK‐rhinitis study	[66]	Obs, CS	3260	NA	20
ARIA score	[67]	Obs, CS	3260	NA	20
Phenotype of allergic diseases and asthma					
Daily allergic multimorbidities	[68]	Obs, CS	4210	32,585	19
Real‐world data in allergic rhinitis					
Treatment of AR using mobile technology with real‐world data: The MASK observational pilot study	[69]	Obs, CS	2871	39,634	25
Mobile technology offers novel insights on control and treatment of AR. The MASK study	[70]	Obs, CS	9122	112,054	23
Impact of allergic diseases					
Work productivity	[71]	Obs, CS	14,189		18
EQ‐5D		Obs, CS			18
WPAI‐AS	[27]	Obs, CS			18
Transfer of innovation	[65]				

Abbreviations: AR, Allergic rhinitis; CS, cross‐sectional study; NA, not applicable; Obs, observational study; WPAI‐AS, Work Productivity and Activity questionnaire.

### 7.4 Step 4: Communicate the Vision. Large Global Dissemination

The coalition has already been very active in promoting ARIA and MASK‐air data and will continue to have a global dissemination.

The integration of new paths of understanding health and change is a requirement for the strategy. A central target is the general need to raise the level of health literacy in society. The general public should clearly not be perceived simply as ‘patients waiting for something to happen’. They should have the ability to navigate and understand health messages, an essential tool for self‐managing wellbeing, even before any condition or major challenge actually occurs. But to do so, one must consider how to improve this health literacy by integrating it much better into the educational system and cultural settings to which it applies. Using AI, we were able to obtain unbiased information on the patient's vision on AR and their needs [48].

### 7.5 Step 5: Empower Others to Act on the Vision

Organisational processes and structures are in place and are aligned with the overall organisational vision. However, we need to continuously check for barriers and for those who are resistant to change, and focus on the education of all stakeholders on how to achieve the best outcomes of prevention treatment. We are acting proactively to remove the obstacles involved in the process of change.

### 7.6 Step 6: Create Short‐Term Wins

Short‐term wins had already been achieved in the 2019 proposal to embed digital data in CM (Table 1) [22]. Data published in the paper [20] can be considered as the short‐term wins of CM2 (Table 2).

**TABLE 2:** Innovative results found in MASK‐air.

1. Allergic rhinitis When the full database is analysed, most patients: are rarely adherent [72] and do not follow guidelines use as‐needed treatment [73, 74] often use OTC medications do not take medication when they are well [73, 74] increase their treatment based on symptoms similarly in all European countries [73] irrespective of the culture [75] and the treatments bought in the pharmacies [73, 76] do not use the recommended treatment Furthermore: Control (symptoms, work productivity, educational performance) is not necessarily improved by increasing medications including co‐medication [69, 70, 73, 77, 78] Co‐medication of an intra‐nasal and an oral medication is usually associated with worse control than single medication (cross‐sectional and longitudinal analyses) [69, 70, 73, 77, 78] Co‐medication is often associated with impaired satisfaction on the treatment [34] Medication satisfaction is reduced on days with co‐medication by comparison to monotherapy Overall, patients treat themselves when they are not well, self‐medicate and increase their medications when they are not controlled, showing that they do not follow guidelines and physician's prescriptions Costs are increased with poor rhinitis control and asthma and rhinitis multimorbidity [79] When adherent patients to the app are analysed: They are also often adherent to medications, and adherence to the app appears to be associated with adherence to medications Except for INCS for which increased adherence is not associated with an increased level of control In patients with an adherence level over 80%, INCS are more effective than AzeFlu for nasal but not ocular symptoms. For an adherence level under 80%, AzeFlu is more effective Most studies were carried out in cross‐sectional studies that were confirmed by longitudinal studies [80] Switching of medications is common (longitudinal analyses) [80] A CSMS has been validated under the auspices of EAACI and ARIA [24]. A monetary value has been ascribed to CSMS [81] 2. Allergen‐specific immunotherapy Evidence regarding the efficacy of allergen immunotherapy (AIT) on AR has been provided mostly by RCTs. A MASK‐air pilot study showed that AIT improves symptoms and work productivity [82] AIT was more effective on days with oral antihistamines (OAH) than on those with intranasal corticosteroids (INCS, monotherapy or comedication) [78] Using a Bayesian model, sublingual was found to be more effective than subcutaneous immunotherapy on VAS symptoms, VAS work and CSMS [83] AIT is likely to have a disease‐modifying effect in contradistinction to pharmacotherapy as shown by the effects on education [84] MASK‐air was considered as a PCC for AIT [33] 3. Asthma When adherent patients to the app are analysed (longitudinal studies): Adherence to ICS or ICS‐LABA was high (56.3%–81.2% days) Adherence was higher for ICS + other LABA than for ICS + formoterol (ICS + F). However, control was similar in both groups, ICS + F was associated with a meaningful lower SABA use and similar VAS asthma, e‐DASTHMA or VAS work Overall, increased adherence to either ICS + F or ICS + other LABA was associated with decreased SABA use For a drug or a drug class, co‐medication days were less well controlled than days with single medication (fixed dose combination therapy considered as single medication) A longitudinal observational study was carried out on asthmatic patients who filled at least 26 days the first month of reporting the MASK‐air app and received a treatment with ICS or ICS/LABA. Based on control, adherence to ICS ± LABA, short‐acting ß‐agonist use and biologics as well as long‐acting anti‐muscarinics, six distinct groups of different clinical relevance were retrieved in non‐switchers Severe asthma Applicability of the MASK‐air app was explored in severe asthmatic patients in a longitudinal (observational) study. Highly variable trends were found for VAS asthma and asthma remission can be assessed using daily e‐DASTHMA and VAS asthma e‐DASTHMA has been validated under the auspices of EAACI and ARIA [25]. A monetary value has been ascribed to e‐DASTHMA [81] Risk factors: Info‐epidemiology studies have been carried out to assess risk factors for asthma exacerbations and can be added to MASK‐air (e.g., rhinovirus [85]). Pollen and pollution are already embedded in MASK‐air for Europe [28, 86, 87]

### 7.7 Step 7: Build on the Change

The current status of MASK‐air 2024–2025 is presented below.

#### 7.7.1 General Data Protection Regulation (GDPR) and Medical Device Regulation (MDR)

The download and usage of the app are free of charge and there are no adverts. It falls under the French jurisdiction and follows the GDPR regulating the processing of personal data in the European Union (EU) [88]. Geolocation also follows the GDPR [89]. The Privacy Impact Assessment (PIA) has been performed and there is an external Data Protection Manager (DPO).

MASK‐air was CE1 registered. However, considering the intended use and the software, MASK‐air became a class IIa medical device (UDI Mask Air app: (01)08720165943005(10)3a0808(11)220600) [90, 91].

#### 7.7.2 The MASK‐Air App Is Based on Patient‐Reported Outcome Measures (e‐PROMs)

Patient‐reported outcome measures (PROMs) are used to assess a patient's health status at a particular point in time and are essential in developing person‐centred care. All MASK‐air VASs are based on PROMs for assessing R, C and A control. VASs were initially developed on paper and pencil and tested for their criterion validity, cut‐offs and responsiveness. Then, a multicentric multinational DB‐PC‐RCT using an electronic VAS form was performed.

VAS for R, A or C were adapted to the digital format in MASK‐air and further methodologic evaluations were performed [92, 93]. VAS work [27, 67, 71, 94] EQ‐5D [27, 67] (VAS and utilities), Work Productivity and Activity Impairment Allergic Specific (WPAI:AS) questionnaire [27, 67, 71, 95] and CARAT [96] are included in the app. For construct validity, in patients with R and/or A, the VASs used were highly correlated to RQLQ and TNSS. Ability to detect a change was also correlated with TNSS or TSS and RQLQ.

Additionally, two control‐medication scores for allergic symptoms of allergy developed with EAACI (Task Forces or Position Papers) (CSMS) or A (e‐DASTHMA) were validated for their criterion validity, cut‐offs and responsiveness. The CSMS [24] displayed similar results in different countries showing the transferability and cultural adaptation of MASK‐air. e‐DASTHMA [25] was strongly correlated with VAS dyspnoea. An external validation was performed using an independent cohort of patients with physician‐diagnosed asthma (INSPIRERS) [97]. e‐DASTHMA was strongly correlated with the GINA classification of control.

#### 7.7.3 Patient Acceptability of MASK‐Air

Two qualitative studies were carried out by MADOPA (*Maintien en Autonomie à Domicile des Personnes Agées*, https://www.madopa.fr) in 2016 to better understand the patients' needs and expectations [30]. Their comments were embedded in MASK‐air.

Five studies carried out in France (in preparation), Italy [98], Lithuania [99], Poland [100] and Portugal [101] showed that patients have an overall positive appreciation of MASK‐air and proposed further improvements of the app. MASK‐air can be used from adolescence [102] to elderly people (up to 85 years of age) [29].

#### 7.7.4 Innovative Results Obtained With MASK‐Air

Several studies have been carried out to better understand the patient's views and behaviour on A and R (Table 2).

**FIGURE 1:**
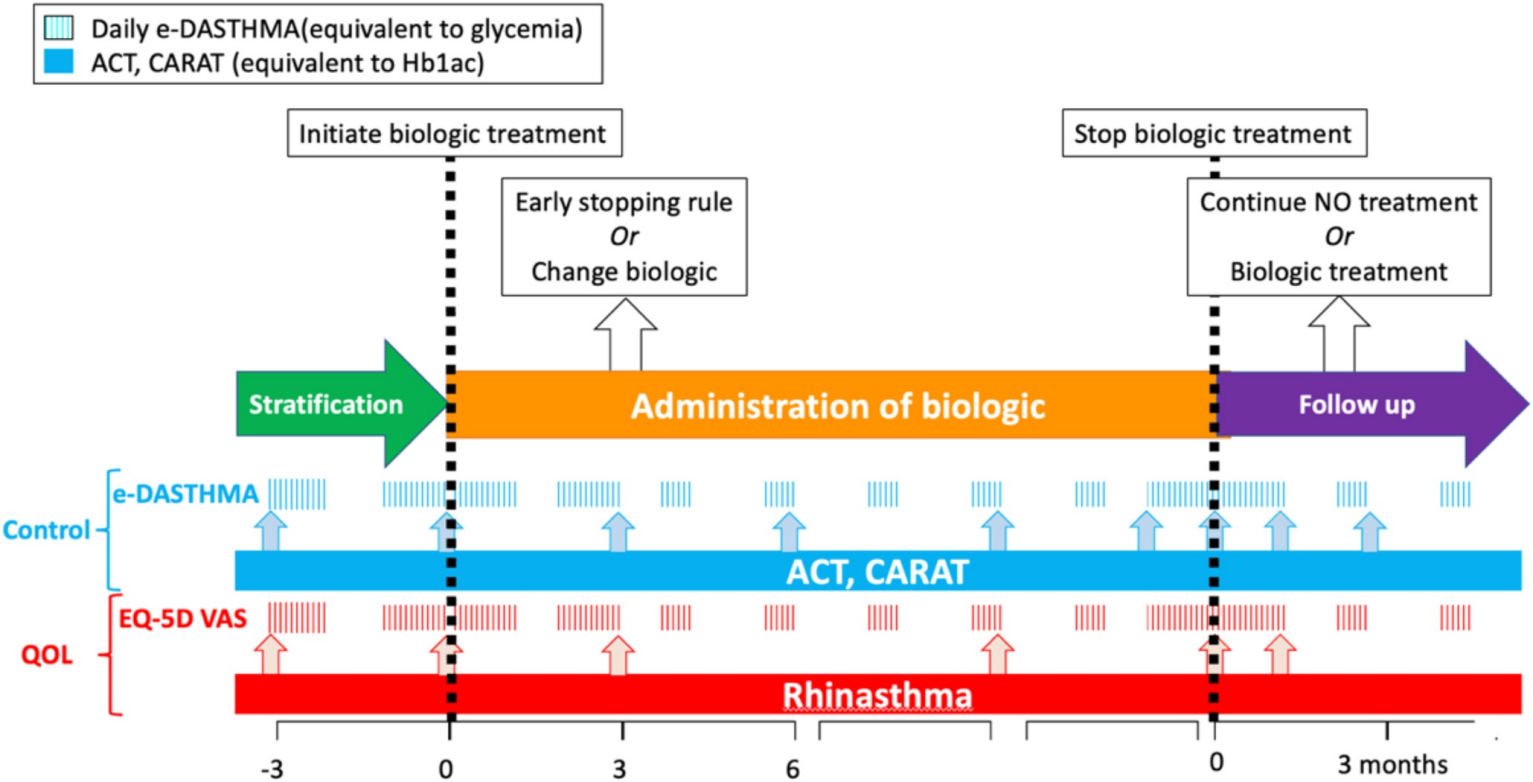
Applicability of digital biomarkers in the treatment of severe asthma by biologics using the diabetes approach (from [32]).

It is proposed that MASK‐air may be relevant in (i) the stratification of severe patients for AIT or biologic treatment assessing the stratification of patients before treatment, (ii) the follow‐up of patients during treatment including the onset of treatment efficacy, (iii) early stopping rules in those with little efficacy, (iv) late stopping rules and finally (v) assessing the relapse of the disease after the cessation of treatment (Figure 1). MASK‐air uses both daily electronic control‐medication scores (CSMS or e‐DASTHMA) and a 1‐month overall control score such as ACT or CARAT.

#### 7.7.5 Impact of Rhinitis and Asthma

MASK‐air data have been used to assess the impact of R, C and/or A using work productivity and education, and high correlations have been observed between VAS work, VAS education [84] or WPAI‐AS [103] and PROMs VAS.

The economic evaluation has been assessed and several MASK‐air tools compared. They include (i) the cost of medications effectively used, (ii) the cost of absenteeism and presenteeism (VAS Work, WPAI‐AS‐work) [104], (iii) the impact on academic productivity [84], (iv) costs of health resource utilisation (EQ‐5D VAS) [23, 104, 105] and (v) potential benefits of expensive treatments such as AIT or biologics. Combining the results of these tools, a monetary value has been ascribed to CSMS and e‐DASTHMA [81].

#### 7.7.6 Environmental Exposure and Planetary Health

**FIGURE 2:**
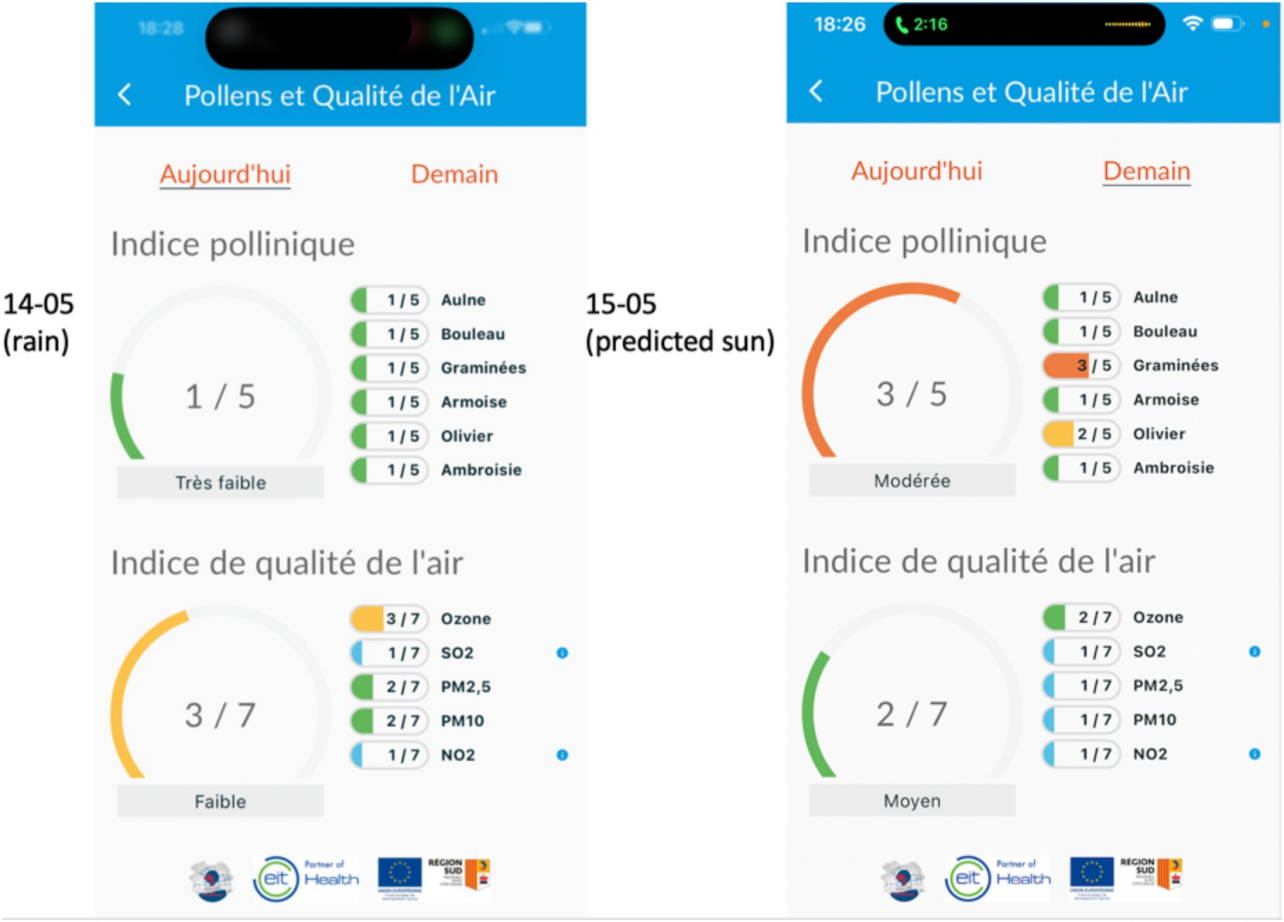
MASK‐air screens on pollen and pollution on two consecutive days during the 2024 grass pollen season in Montpellier, France.

In Europe, MASK‐air is combined with prediction on allergen exposure and air quality (POLLAR: Impact of POLLution on Asthma and Rhinitis, EIT Health‐funded project) using COPERNICUS data (Figure 2) [86].

In December 2019, a conference entitled ‘Europe That Protects: Safeguarding Our Planet, Safeguarding Our Health’ was held in Helsinki. It was co‐organised by the Finnish Institute for Health and Welfare, the Finnish Environment Institute and the European Commission, under the auspices of Finland's Presidency of the EU [106, 107]. As a side event, a symposium organised as the final POLLAR meeting explored the digital transformation of health and care to sustain planetary health in airway diseases [56]. The Finnish Allergy Programme [108] collaborates with MASK‐air and can be considered as a proof‐of‐concept to impact Planetary Health.

### 7.8 Step 8: Anchor the Changes in Corporate Culture

Building an alliance among patients, healthcare providers and policy makers is therefore essential to save healthcare costs and provide better care for the patients. Healthcare providers or insurers could give a financial reward for patients with chronic disease using digital health tools or HCPs. It is of upmost importance to transfer the knowledge of any person‐centred care approach to policy makers.

#### 7.8.1 Change Management in ARIA 2024–2025 Guidelines

ARIA has evolved from a guideline using the best evidence‐based approach (Grading of Recommendations, Assessment, Development and Evaluation: GRADE) [53] to integrated care pathways using mobile technology in patients with allergic rhinitis (AR) and A multimorbidity [109].

**FIGURE 3:**
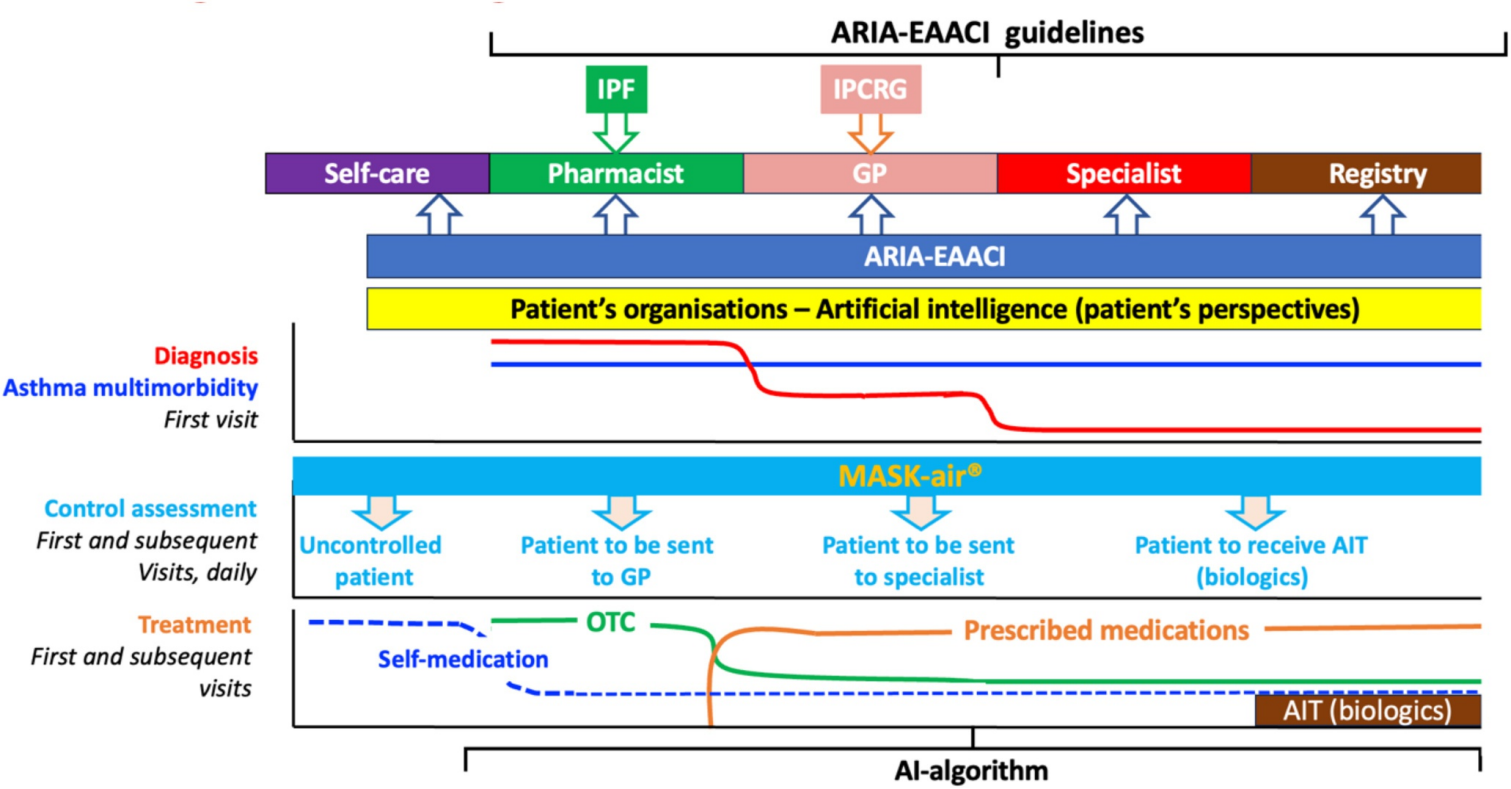
Person‐centred, digitally enabled, AI‐assisted care pathways based on ARIA‐EAACI guidelines using MASK‐air as a common tool: OECD Best Practice. AI, artificial intelligence; AIT, allergen immunotherapy; IPCRG, International Primary Care Respiratory Group; IPF, International Pharmacist Federation; OTC, over‐the‐counter.

The current knowledge to develop the next iteration of ARIA (ARIA 2024–2025) includes [63] (i) person's values and preferences to guide clinical decisions [110], (ii) digital care pathways for AR + A multimorbidity based on PROMs using GRADE as the evidence‐based model [111, 112, 113, 114, 115, 116, 117], (iii) digitally enabled person‐centred care [17] combining all relevant research evidence including so‐called real‐world evidence (MASK‐air data), (iv) distinguishing patients with AR alone from those with AR + A, (v) AI that was essential in developing (patient/population, intervention, comparison and outcomes) questions and recommendations on real‐world data to triangulate RCTs questions [48], (vi) considering planetary health [118], (vii) including asthma in the recommendations and (viii) embedding Planetary Health (Figure 3).

#### 7.8.2 Best Practice of OECD on Integrating Care to Prevent and Manage Chronic Diseases

In the recent report of the Organisation for Economic Coordination and Development (OECD) on Best Practices (BPs) for Integrating Care to Prevent and Manage Chronic Diseases, the MASK‐air app on rhinitis and asthma (MASK‐air) has been listed [18]. The OECD is a reliable source of evidence‐based policy analysis and economic data largely used by governments. It has published several BPs on Public Health. On 10 May 2023, the OECD published 13 BPs for Integrating Care to Prevent and Manage Chronic Diseases in the European Union. The report did not cover all models of integrated care, rather, it ‘focuse(d) on those that are of key strategic interest to policy makers’. The ARIA‐EAACI CM is presented in the OECD BP.

#### 7.8.3 Governmental Endorsement

Currently, the Polish and Ukrainian governments have endorsed MASK‐air. The Japanese government has granted MASK‐air for its deployment in Japan.

#### 7.8.4 Equity

A special effort needs to be made to globalise care pathways and make them applicable to all patients [119]. The first ARIA report already involved low‐ and middle‐income countries [10]. MASK‐air can be used in all countries since it uses simple PROMs in different cultural environments. A specific group of members of developing countries is involved in ARIA 2024.

Smartphone ownership is growing rapidly around the world. In 2015, there were more than 7 billion mobile telephone subscriptions across the world, over 70% of which were in low‐ or middle‐income countries [120]. The joint WHO‐ITU (International Telecommunication Union) initiative ‘Be He@lthy, Be Mobile’ for the prevention and management of noncommunicable diseases, their comorbidities and their risk factors, including improving disease diagnosis and tracking, is of great importance. MASK is one of the examples of the ‘Be He@lthy, Be Mobile’ handbook on how to implement mBreatheFreely for asthma and COPD [121] and is a demonstration project of GARD [64].

OECD has specifically identified MASK‐air as an equity‐enhancing app since it is available in 30 countries. It also pointed out that MASK‐air ‘offers the same level of care for AR and asthma to people across the world’.

#### 7.8.5 UCRAID: Ukrainian Citizen and Refugee Electronic Support in Respiratory, Allergy, Immunology and Dermatology Action Plan

An estimated 6 to 7 million Ukrainians have taken refuge in the European Union. Many have A and/or AR and/or urticaria, and around 100,000 may have a severe disease. Cultural and language barriers are a major obstacle to appropriate management. Two mHealth apps—MASK‐air (Mobile Airways Sentinel networK), for the management of R and A and CRUSE (Chronic Urticaria Self Evaluation) [122] for urticaria patients—are available in many countries and languages [62]. The Ukrainian patients fill in the questionnaires and daily symptom‐medication scores for A and/or A (MASK‐air) or urticaria (CRUSE) in Ukrainian. Following the GDPR, patients grant their physician access to the app by scanning a QR code displayed on the physician's computer, enabling the physician to read the app contents in his/her own language. This service is available freely. It takes less than a minute to show patient data to the physician on the physician's web browser. UCRAID is under the auspices of the Ministry of Health of Ukraine.

### 7.9 Conclusion

The potential benefits of MASK‐air as an app or as a tool using MDR Class IIa (MASK‐air‐Pro) are listed in Table 3.

**TABLE 3:** Global applicability of MASK‐air.

Applicability	MASK‐air
MASK includes an app (MASK‐air) that can be used as a Medical Device (MDR Class IIa: MASK‐air‐Pro)	
Major MASK‐air findings	The results of MASK‐air indicate that many patients are uncontrolled and non‐adherent to treatment Patients appear to use their medications as needed and not as a regular basis as prescribed They are not always satisfied with their treatment
Patient empowerment	*Self‐management*: The patient takes control of his(her) own disease to improve autonomy due to: Better understanding of the symptoms Aerobiology (pollen and pollution) data embedded in control Improved adherence *MASK‐air as a medical device (MDR class IIa)* has the potential to link patients, counselling (messaging and warnings) and physician's advice
Clinical practice	MASK‐air is an essential tool for providing *personalized medicine* in AR and asthma *Optimized shared‐decision making*. Physicians will be able to read the daily files of the patients in order to: ⚬ Assess exacerbations and their link with pollen and pollution ⚬ Assess and increase the adherence to treatment ⚬ Assess daily patient's satisfaction to the treatment ⚬ Optimize treatment for the patient and, in particular, the current or the next pollen season Prescribe allergen immunotherapy (AIT) more rapidly when the patient is not controlled despite optimal pharmacologic treatment and determine AIT efficacy Assess and manage severe asthma
Registries	Daily symptom‐medication scores, adherence and satisfaction are of great interest for registries MASK‐air may become the link between different registries (common tool used in different languages)
Cost‐effectiveness	Several tools in MASK‐air can be used for costs (work productivity, WPAI‐AS, daily medication costs) and utilities (EQ‐5D) Identify the most effective treatment strategy based on real‐life data, taking into account adherence and treatment persistence The use of real‐life data from MASK‐air provides added value by making the pharmaco‐economic simulation more accurate and reliable
Clinical trials	*For RCTs*, it is essential to have clarity on definitions and relevant tools. MASK‐air allows: ⚬ Better stratification of the patients needing treatment ⚬ Assessment of the onset and magnitude of treatment efficacy during the trial ⚬ Assessment of the efficacy when the treatment is stopped *Observational studies* in real life: ⚬ Are of key importance to triangulate RCTs ⚬ Can compare different treatment strategies ⚬ Provide insight into delivery of care in routine practice to all patients ⚬ Can provide answers to questions that have not or cannot be evaluated in RCTs, especially effectiveness on a longer timescale ⚬ Can reflect changes of attitude to treatment over time in a rapidly changing overall medical landscape ⚬ Can include cost‐effectiveness ⚬ Bring new hypotheses for the treatment of AR and asthma
Registration and reimbursement of treatments	Controlled trials designed with a uniform approach will be more easily evaluated by the Health Technology Assessment agencies (such as NICE) for reimbursement. MASK‐air uses EQ‐5D, a validated measure of utility Better understanding of direct and indirect costs
Public health planning	For public health purposes, a perfect patient characterization in real life is needed to identify the prevalence, burden and costs incurred by patients in order to improve quality of care and optimize healthcare planning and policies.
Reduction of inequities	Inequities still exist in the EU for the prevalence and burden of allergic diseases (not only sex/gender inequities). In deprived areas, asthma is often severe OECD specifically identified MASK‐air as an equity‐enhancing app
Employers	AR and asthma represent a major burden for the employers, and the estimated annual costs in the EU range from 30 to 60 B€. Better control of the disease was shown to reduce costs. MASK‐air has the potential to improve the control of allergic diseases and asthma and to significantly improve work productivity at the EU level MASK‐air as an MDR class IIa may help to propose and implement preventive strategies of occupational diseases
Guideline development	Real‐life data of MASK‐air represent an asset for ARIA 2024
Research on mechanisms and genetics	A uniform definition and a collaborative approach to research including genetic and mechanistic research are important and will be enhanced by the stratification of patients using MASK‐air Different levels of phenotype characterization (granularity) can be applied to assess phenotypic characterization in different groups of patients (e.g., rhinitis alone and rhinitis and asthma) including gender and old age patients
Epidemiology	In epidemiologic population studies, standardized definitions and tools are fundamental. MASK‐air allows novel approaches combining classical cross‐sectional and longitudinal studies with real‐life studies in large populations MASK‐air may become the link between different epidemiologic studies
Humanitarian actions	UCRAID: Ukrainian Citizen and Refugee Electronic Support in Respiratory, Allergy, Immunology and Dermatology action plan From UCRAID to travels
From MASK‐air to symptomatic chronic diseases	The MASK approach is a prototype of digital health for symptomatic chronic diseases without objective measurements (e.g., arterial pressure, glycaemia)

## 8 The Future

### 8.1 Optimisation of the Tools

#### 8.1.1 Overall Limitations and Considerations for Practical Implementation

mHealth apps can result in improvements of care in patients with R and/or A. However, to maximise the clinical impact of MASK‐air, they should not be considered ‘one size fits all’ solutions and their overall limitations should be taken into account. First, some participants may not be able to use mHealth apps (for instance, they may not own a smartphone or have low digital literacy) [98, 101, 123]. Among those participants, older patients and patients with a lower income may be under‐represented. In addition, without regular encouragement from their physicians, participants may keep using mHealth apps for only short periods of time. The active involvement of physicians in promoting the use of mHealth apps in clinical consultations was found to increase the duration of reporting (Sousa‐Pinto, personal communication).

In MASK‐air, two groups of patients fill the app. Those who use the app for one to a few days and those who are adherent to the app and use it for over 90 days as a mean. With patient's organisations, there is a need to develop tools to help more patients use the app for longer periods.

#### 8.1.2 Environmental Exposure, Climate Change and Planetary Health

A new Horizon Europe grant started in September 2022 in order to better understand climate change and how to counteract it. Pollens were selected as one indicator of climate change. In collaboration with the Finnish Meteorological Institute (FMI) and Porto University, MASK‐air will be used to correlate pollen counts with the clinical impact (EU Horizon Europe grant Catalyse). For this project, preliminary methodologic studies are undergoing that will help to better assess the impact of the pollen season on symptoms [124]. The integration of information technology tools for climate, weather, air pollution and aerobiology in MASK‐air will enable the development of an alert system. Citizens will thus be informed about personal environmental threats, which may also be linked to indicators of Planetary Health and sustainability.

#### 8.1.3 Costs of Interventions

MASK‐air data can also inform about the impact of the disease using quality‐of‐life (EQ‐5D‐5L [23]), activities (WPAI‐AS [106]), work productivity and education (VAS work [21, 92, 94, 125] and education [84], WPAI‐AS [86]).

The economic evaluation is currently being assessed and several MASK‐air tools can be compared. Evaluating the cost of an intervention is intrinsically linked to assessing its effectiveness. We can justify incremental expenses if they are cost‐effective. They include at least (i) the cost of medications effectively used and (ii) the cost of absenteeism and presenteeism (VAS Work, WPAI‐AS‐work) [103]. By combining data from these tools with the treatment effect, we can evaluate any treatment in comparison to others. This approach helps identify the most effective treatment strategy based on real‐life data, also taking into account adherence and treatment persistence. Using real‐life data from MASK‐air provides added value by making the pharmaco‐economic simulation more accurate and reliable.

### 8.2 Implementation in Low‐ and Middle‐Income Countries

In the first ARIA guideline in 1999, a large section was devoted to developing countries in collaboration with IUTLD (International Union of Tuberculosis and Lung Diseases) [10]. Then, ARIA was a GARD member and all its iterations included implementation in low‐ and middle‐income countries (LMICs) [64]. The MASK‐air app was designed to be used in LMICs and has been launched in middle‐income countries such as Albania, Brazil, Ecuador, Lebanon, Mexico and Serbia. MASK‐air has been listed as one of the apps of the ‘Be He@lthy, Be Mobile’ handbook on asthma and COPD (WHO‐ITU [121]).

The aim is to develop a coalition to deploy and test MASK‐air in LMICs since the app is available in Arabic, French, English, Portuguese and Spanish. The only requirement is the use of a smartphone. Despite increases in smartphone ownership, many emerging economies still trail advanced economies. The ultimate aim of the project is to strengthen WHO on harnessing the power of digital technologies and health innovation to accelerate global attainment of health and well‐being [126].

### 8.3 Transformation of Health and Care

An overall approach for a person‐centred, digitally enabled, AI‐assisted care pathway should include all stakeholders in a stepwise and coordinated approach from self‐care to the management of severe diseases and registries (Figure 3). An mHealth tool such as MASK‐air can provide an easy link between patients and all care practitioners. The ARIA‐EAACI guidelines for allergic rhinitis can be used to optimise management in a cost‐effective manner.

#### 8.3.1 In Rhinitis: Allergen Immunotherapy and Biologics

The importance of registries in AIT has been identified. There is a huge need to understand the effects of AIT on patient outcomes. Although clinical research trials have shown benefit, it is important to also see this when treating patients in the ‘real world’. Many registries are simply a place to put data. For others, like the BSACI registry (BRIT), there are many direct benefits for patients and clinicians (https://www.bsaci.org/professional‐resources/bsaci‐registries/immunotherapy‐registry/#:~:text=The%20registry%20enhances%20patient%20safety,take%20place%20during%20immunotherapy%20treatment).

Digital tools are promoters for PCC practices in chronic care [7]. Alone, they cannot achieve the genuine aims of PCC [8] but, when combined to a registry, mHealth app data appear to be optimal for a PCC approach.

Some biologics have been used in severe uncontrolled rhinitis patients. Due to their costs, approved registries will be needed to better assess their cost‐effective relevance.

#### 8.3.2 The Diabetes Approach in Asthma

In diabetes, two types of biomarkers are defined to monitor disease control [127, 128]: the daily control monitoring (glycaemia measurement) and longer‐term monitoring (glycated haemoglobin (HbA1c) measurement). Both tests are required to optimise diabetes management. By analogy with the diabetes approach, two types of patient‐centred digital biomarkers are available for R and A:

- Long‐term monitoring using control scores using CARAT (analogous to HbA1C measurement) [96, 129, 130]: this is proposed as it combines both R and A control with a recall period of 4 weeks, whereas other rhinitis (e.g., Allergic Rhinitis Control Test [131], Rhinitis Control Assessment Test [132]) or asthma (e.g., Asthma Control Questionnaire—ACQ [133]) control questionnaires are based on a 1‐week recall period. The Asthma Control Test—ACT is based on a 4‐week recall [134]. These questionnaires, however, do not fully capture the control in patients with fluctuating symptoms (particularly those with severe asthma).
- Daily monitoring of the control (analogous to glycaemia measurement): this can be measured daily using the CSMS [24] for allergy or e‐DASTHMA for asthma [25].

#### 8.3.3 Severe Asthma

Although the efficacy of biologics in severe asthma has been demonstrated, their cost‐effectiveness is still questionable both in high‐income [135, 136] and middle‐income countries [137, 138]. An adequate cost‐effective choice of these costly and long‐lasting therapies is thus needed [139, 140]. The introduction of biologics in severe asthma therefore increases the need for biomarkers in patient selection, prediction of outcomes and monitoring (Figure 3). Genetic or biologic biomarkers are not yet able to be used in routine clinical practice globally. Digital biomarkers may therefore provide a personalised approach for optimal person‐centred shared‐decision making in the stratification and follow‐up of patients [32, 141, 142, 143].

Currently, a major criterion to initiate or stop a biologic in asthma is the frequency of exacerbations. However, this is vague and an optimised patient stratification is needed. The MASK‐air app has potential with regards to public health care, defining lack of adherence, medication satisfaction, lack of control with huge potential for optimisation of healthcare costs. Finally, MASK‐air may be introduced into public payer systems in SA.

## 9 Conclusion

The second CM in airways diseases (ARIA‐EAACI 2025) extends largely beyond a guideline. (i) It is person‐centred and not simply patient‐centred as it includes more than a clinical perspective. (ii) It uses a validated mHealth tool (MASK‐air) approved by governments and OECD that represents ‘a strategically‐important health care model …. confirmed using a validated performance assessment [OECD] ….. [that] is of strategic interest to policy makers’ [18]. (iii) It is AI‐assisted, particularly for patients' views and expectations, including values and preferences [110, 144]. (iv) Newly identified phenotypes of airway diseases are also considered. It is leading to the ARIA 2024–2025 guideline that includes major novel steps in the GRADE guidelines such as embedding real‐life data or planetary health. This CM strategy may be used as a model for other chronic diseases following the initial proposal for MASK‐air in 2015 [145]. It also has the strength to be built and implemented with EAACI.

## Author Contributions

**Jean Bousquet:** conceptualization, funding acquisition, writing – original draft, methodology, validation, writing – review and editing, formal analysis, supervision. **Bernardo Sousa‐Pinto:** conceptualization, investigation, funding acquisition, methodology, validation, writing – review and editing, visualization, formal analysis. **Mohamed H. Shamji:** conceptualization, methodology, validation, writing – review and editing. **Maria J. Torres:** conceptualization, methodology, validation, writing – review and editing. **Ludger Klimek:** conceptualization, validation, writing – review and editing, methodology. **Holger J. Schünemann:** methodology, writing – review and editing, conceptualization, validation. **Mario Morais‐Almeida:** conceptualization, writing – review and editing. **Rafael José Vieira:** conceptualization, methodology, validation, writing – review and editing. **Alkis Togias:** conceptualization, methodology, writing – review and editing, validation. **Boleslaw Samolinski:** conceptualization, methodology, validation, writing – review and editing. **Arunas Valiulis:** conceptualization, methodology, validation, writing – review and editing. **Siân Williams:** conceptualization, methodology, validation, writing – review and editing. **Oliver Pfaar:** conceptualization, methodology, validation, writing – review and editing. **Torsten Zuberbier:** conceptualization, methodology, validation, writing – review and editing. **Anna Bedbrook:** writing – review and editing, project administration. **Wienczyslawa Czarlewski:** conceptualization, writing – review and editing. **Maryam Ali Al‐Nesf:** conceptualization, methodology, validation, writing – review and editing. **Rita Amaral:** conceptualization, methodology, validation, writing – review and editing. **Josep M. Anto:** conceptualization, methodology, validation, writing – review and editing. **Antonio Bognanni:** conceptualization, methodology, validation, writing – review and editing. **Luisa Brussino:** conceptualization, methodology, validation, writing – review and editing. **Alvaro A. Cruz:** conceptualization, methodology, validation, writing – review and editing. **Violeta Kvedariene:** conceptualization, methodology, validation, writing – review and editing. **Habib Douagui:** conceptualization, methodology, validation, writing – review and editing. **Nikolaos G. Papadopoulos:** conceptualization, methodology, validation, writing – review and editing. **G. Walter Canonica:** conceptualization, methodology, validation, writing – review and editing. **Ivan Cherrez‐Ojeda:** conceptualization, methodology, validation, writing – review and editing. **Mark Dykewicz:** conceptualization, methodology, validation, writing – review and editing. **Bilun Gemicioglu:** conceptualization, methodology, validation, writing – review and editing. **Mattia Giovannini:** conceptualization, methodology, validation, writing – review and editing. **Brigita Gradauskiene:** conceptualization, methodology, validation, writing – review and editing. **Tari Haahtela:** conceptualization, methodology, validation, writing – review and editing. **Cristina Jacomelli:** conceptualization, methodology, validation, writing – review and editing. **Tuomas Jartti:** conceptualization, methodology, validation, writing – review and editing. **Miloš Jeseňák:** conceptualization, methodology, validation, writing – review and editing. **Piotr Kuna:** conceptualization, methodology, validation, writing – review and editing. **Désirée E. Larenas‐Linnemann:** conceptualization, methodology, validation, writing – review and editing. **Amir H. A. Latiff:** conceptualization, methodology, validation, writing – review and editing. **Bryan Martin:** conceptualization, methodology, validation, writing – review and editing. **Yousser Mohammad:** conceptualization, methodology, validation, writing – review and editing. **Kari Nadeau:** conceptualization, methodology, validation, writing – review and editing. **Elizabete Nunes:** conceptualization, methodology, validation, writing – review and editing. **Ken Ohta:** conceptualization, methodology, validation, writing – review and editing. **Martial Ouédraogo:** conceptualization, methodology, validation, writing – review and editing. **Padukudru A. Mahesh:** conceptualization, methodology, validation, writing – review and editing. **Isabella Pali‐Schölll:** conceptualization, methodology, validation, writing – review and editing. **Ana Margarida Pereira:** conceptualization, methodology, writing – review and editing, validation. **Frederico S. Regateiro:** conceptualization, methodology, validation, writing – review and editing. **Nicolas Roche:** conceptualization, methodology, validation, writing – review and editing. **Mikhail Sofiev:** conceptualization, methodology, validation, writing – review and editing. **Luis Taborda‐Barata:** conceptualization, methodology, writing – review and editing, validation. **Charlotte Suppli Ulrik:** conceptualization, methodology, validation, writing – review and editing. **Sanna K. Toppila‐Salmi:** conceptualization, methodology, validation, writing – review and editing. **Marylin Valentin Rostan:** conceptualization, methodology, validation, writing – review and editing. **Leticia de las Vecillas:** conceptualization, methodology, validation, writing – review and editing. **Maria Teresa Ventura:** conceptualization, methodology, validation, writing – review and editing. **Giovanni Viegi:** conceptualization, methodology, validation, writing – review and editing. **De Yun Wang:** conceptualization, methodology, validation, writing – review and editing. **He Zhang:** conceptualization, methodology, validation, writing – review and editing. **Luo Zhang:** conceptualization, methodology, validation, writing – review and editing. **Giorgio Ciprandi:** conceptualization, methodology, validation, writing – review and editing. **Juan Carlos Ivancevich:** conceptualization, methodology, validation, writing – review and editing. **Nikolai Khaltaev:** conceptualization, methodology, validation, writing – review and editing. **Olga Lourenço:** conceptualization, methodology, validation, writing – review and editing. **Lucas Leemann:** conceptualization, methodology, validation, writing – review and editing. **Marine Savouré:** conceptualization, methodology, validation, writing – review and editing. **Juan Jose Yepes‐Nuñez:** conceptualization, methodology, validation, writing – review and editing. **Arzu Yorgancioglu:** conceptualization, methodology, validation, writing – review and editing. **Baharudin Abdullah:** conceptualization, methodology, validation, writing – review and editing. **Mona Al‐Ahmad:** conceptualization, methodology, validation, writing – review and editing. **Julijana Asllani:** conceptualization, methodology, validation, writing – review and editing. **Karl‐C. Bergmann:** conceptualization, methodology, validation, writing – review and editing. **Jonathan A. Bernstein:** conceptualization, methodology, validation, writing – review and editing. **Michael S. Blaiss:** conceptualization, methodology, validation, writing – review and editing. **Fulvio Braido:** conceptualization, methodology, validation, writing – review and editing. **Pedro Carreiro‐Martins:** conceptualization, methodology, validation, writing – review and editing. **Lorenzo Cecchi:** conceptualization, methodology, validation, writing – review and editing. **Antonio F. M. Giuliano:** conceptualization, methodology, validation, writing – review and editing. **George Christoff:** conceptualization, methodology, validation, writing – review and editing. **Ieva Cirule:** conceptualization, methodology, validation, writing – review and editing. **Jaime Correia‐de‐Sousa:** conceptualization, methodology, validation, writing – review and editing. **Elisio M. Costa:** conceptualization, methodology, validation, writing – review and editing. **Biljana Cvetkovski:** conceptualization, methodology, validation, writing – review and editing. **Stefano Del Giacco:** conceptualization, methodology, validation, writing – review and editing. **Philippe Devillier:** conceptualization, methodology, validation, writing – review and editing. **Dejan Dokic:** conceptualization, methodology, validation, writing – review and editing. **Maia Gotua:** conceptualization, methodology, validation, writing – review and editing. **Maria Antonieta Guzman:** conceptualization, methodology, validation, writing – review and editing. **Elham Hossny:** conceptualization, methodology, validation, writing – review and editing. **Tomohisa Iinuma:** conceptualization, methodology, writing – review and editing, validation. **Carla Irani:** conceptualization, methodology, writing – review and editing, validation. **Zhanat Ispayeva:** conceptualization, methodology, writing – review and editing, validation. **Kaja Julge:** conceptualization, methodology, writing – review and editing, validation. **Igor Kaidashev:** conceptualization, methodology, validation, writing – review and editing. **Kazi S. Bennoor:** conceptualization, methodology, writing – review and editing, validation. **Helga Kraxner:** conceptualization, methodology, writing – review and editing, validation. **Inger Kull:** conceptualization, methodology, writing – review and editing, validation. **Marek Kulus:** conceptualization, methodology, validation, writing – review and editing. **Maciej Kupczyk:** conceptualization, methodology, validation, writing – review and editing. **Andriy Kurchenko:** conceptualization, methodology, validation, writing – review and editing. **Xin Luo:** conceptualization, methodology, validation, writing – review and editing. **Stefania La Grutta:** conceptualization, methodology, validation, writing – review and editing. **Lan Le Thi Tuyet:** conceptualization, methodology, validation, writing – review and editing. **Michael Makris:** conceptualization, methodology, validation, writing – review and editing. **Branislava Milenkovic:** conceptualization, methodology, validation, writing – review and editing. **Neven Miculinic:** conceptualization, methodology, validation, writing – review and editing. **Sang Min Lee:** conceptualization, methodology, validation, writing – review and editing. **Stephen Montefort:** conceptualization, methodology, validation, writing – review and editing. **André Moreira:** conceptualization, methodology, validation, writing – review and editing. **Joaquim Mullol:** conceptualization, methodology, validation, writing – review and editing. **Rachel Nadif:** conceptualization, methodology, validation, writing – review and editing. **Alla Nakonechna:** conceptualization, methodology, validation, writing – review and editing. **Hugo E. Neffen:** conceptualization, methodology, validation, writing – review and editing. **Stefania Nicola:** conceptualization, methodology, validation, writing – review and editing. **Marek Niedoszytko:** conceptualization, methodology, validation, writing – review and editing. **Dieudonné Nyembue:** conceptualization, methodology, validation, writing – review and editing. **Robyn E. O’Hehir:** conceptualization, methodology, validation, writing – review and editing. **Ismail Ogulur:** conceptualization, methodology, validation, writing – review and editing. **Yoshitaka Okamoto:** conceptualization, methodology, validation, writing – review and editing. **Markus Ollert:** conceptualization, methodology, validation, writing – review and editing. **Heidi Olze:** conceptualization, methodology, validation, writing – review and editing. **Oscar Palomares:** conceptualization, methodology, validation, writing – review and editing. **Petr Panzner:** conceptualization, methodology, validation, writing – review and editing. **Hae‐Sim Park:** conceptualization, methodology, validation, writing – review and editing. **Vincenzo Patella:** conceptualization, methodology, validation, writing – review and editing. **Ruby Pawankar:** conceptualization, methodology, validation, writing – review and editing. **Constantinos Pitsios:** conceptualization, methodology, validation, writing – review and editing. **Todor A. Popov:** conceptualization, methodology, validation, writing – review and editing. **Francesca Puggioni:** conceptualization, methodology, validation, writing – review and editing. **Santiago Quirce:** conceptualization, methodology, validation, writing – review and editing. **Agné Ramonaité:** conceptualization, methodology, validation, writing – review and editing. **Marysia Recto:** conceptualization, methodology, validation, writing – review and editing. **Maria Susana Repka‐Ramirez:** conceptualization, methodology, validation, writing – review and editing. **Karla Robles‐Velasco:** conceptualization, methodology, validation, writing – review and editing. **Menachem Rottem:** conceptualization, methodology, validation, writing – review and editing. **Marianella Salapatas:** conceptualization, methodology, validation, writing – review and editing. **Joaquin Sastre:** conceptualization, methodology, validation, writing – review and editing. **Nicola Scichilone:** conceptualization, methodology, validation, writing – review and editing. **Juan‐Carlos Sisul:** conceptualization, methodology, validation, writing – review and editing. **Dirceu Solé:** conceptualization, methodology, validation, writing – review and editing. **Manuel Soto‐Martinez:** conceptualization, methodology, validation, writing – review and editing. **Milan Sova:** conceptualization, methodology, validation, writing – review and editing. **Katarina Stevanovic:** conceptualization, methodology, validation, writing – review and editing. **Pongsakorn Tantilipikorn:** conceptualization, methodology, validation, writing – review and editing. **Ana Todo‐Bom:** conceptualization, methodology, validation, writing – review and editing. **Vladyslav Tsaryk:** conceptualization, methodology, validation, writing – review and editing. **Ioanna Tsiligianni:** conceptualization, methodology, validation, writing – review and editing. **Marilyn Urrutia‐Pereira:** conceptualization, methodology, validation, writing – review and editing. **Erkka Valovirta:** conceptualization, methodology, validation, writing – review and editing. **Tuula Vasankari:** conceptualization, methodology, validation, writing – review and editing. **Dana Wallace:** conceptualization, methodology, validation, writing – review and editing. **Margitta Worm:** conceptualization, methodology, validation, writing – review and editing. **Osman M. Yusuf:** conceptualization, methodology, validation, writing – review and editing. **Fares Zaitoun:** conceptualization, methodology, validation, writing – review and editing. **Mihaela Zidarn:** conceptualization, methodology, validation, writing – review and editing.

## Funding

The authors have nothing to report.

## Ethics Statement

The authors have nothing to report.

## Conflicts of Interest

Jean Bousquet reports personal fees from Cipla, Menarini, Mylan, Novartis, Purina, Sanofi‐Aventis, Teva, Noucor, other from KYomed‐Innov, other from Mask‐air‐SAS, outside the submitted work.

Oliver Pfaar reports grants and personal fees from ALK‐Abelló, grants and personal fees from Allergopharma, grants and personal fees from Stallergenes Greer, grants and personal fees from HAL Allergy Holding B.V./HAL Allergie GmbH, grants and personal fees from Bencard Allergie GmbH/Allergy Therapeutics, grants and personal fees from Laboratorios LETI/LETI Pharma, grants and personal fees from GlaxoSmithKline, personal fees from ROXALL Medizin, personal fees from Novartis, grants and personal fees from Sanofi‐Aventis and Sanofi‐Genzyme, personal fees from Med Update Europe GmbH, personal fees from streamedup! GmbH, personal fees from Pohl‐Boskamp, grants from Inmunotek S.L., personal fees from John Wiley and Sons, AS, personal fees from Paul‐Martini‐Stiftung (PMS), personal fees from Regeneron Pharmaceuticals Inc., personal fees from RG Aerztefortbildung, personal fees from Institut für Disease Management, personal fees from Springer GmbH, grants and personal fees from AstraZeneca, personal fees from IQVIA Commercial, personal fees from Ingress Health, personal fees from Wort&Bild Verlag, personal fees from Verlag ME, personal fees from Procter&Gamble, personal fees from ALTAMIRA, personal fees from Meinhardt Congress GmbH, personal fees from Deutsche Forschungsgemeinschaft, personal fees from Thieme, grants from Deutsche AllergieLiga e.V., personal fees from AeDA, personal fees from Alfried‐Krupp Krankenhaus, personal fees from Red Maple Trials Inc., personal fees from Königlich Dänisches Generalkonsulat, personal fees from Medizinische Hochschule Hannover, personal fees from ECM Expro&Conference Management, personal fees from Technical University Dresden, grants and personal fees from Lilly, personal fees from Japanese Society of Allergy, personal fees from Forum für Medizinische Fortbildung, personal fees from Dustri‐Verlag, personal fees from Pneumolive, grants and personal fees from ASIT Biotech, personal fees from LOFARMA, personal fees from Paul‐Ehrlich‐Institut, personal fees from Almirall, from BREAZY Health, outside the submitted work; and Vice President and member of EAACI Excom, member of ext. board of directors DGAKI; coordinator, main‐ or co‐author of different position papers and guidelines in rhinology, allergology and allergen‐immunotherapy; Editor‐in‐Chief (EIC) of Clinical Translational Allergy(CTA), Associate Editor (AE) of Allergy.

Maria J. Torres reports personal fees from Leti Laboratories, personal fees from Aimmune Therapeutics, personal fees from Diater Laboratories, grants from European Commission, grants from ISCIII, grants from SEAIC, outside the submitted work.

Maciej Kupczyk reports personal fees from Abbvie, personal fees from Astra Zeneca, personal fees from Almiral, personal fees and non‐financial support from Berlin Chemie, personal fees from Chiesi, personal fees from EMMA, personal fees from HVD, personal fees from Adamed, personal fees from GSK, personal fees from Sanofi, personal fees from Novartis, personal fees from Teva, personal fees from Lek‐AM, personal fees from Celon Pharma, personal fees from HAl Allergy, personal fees from ZentivAa, outside the submitted work.

Ivan Cherrez‐Ojeda reports grants from Universidad Espiritu Santo, outside the submitted work.

Michael S. Blaiss reports personal fees from AstraZeneca, personal fees from GSK, personal fees from Sanofi, personal fees from Regeneron, personal fees from ALK, personal fees from SoundHealth, outside the submitted work.

Stefano Del Giacco reports grants and personal fees from AstraZeneca, personal fees from Chiesi, grants and personal fees from GSK, grants and personal fees from Novartis, grants and personal fees from Sanofi, personal fees from CSL‐Behring, personal fees from Takeda, outside the submitted work.

Nikolaos G. Papadopoulos reports grants from Nestle, Numil, Vianex, Vibrant, personal fees from Abbott, Astra Zeneca, GSK, HAL, Menarini, Novartis, Danone Nutricia, OM Pharma, Regeneron, Sanofi, outside the submitted work.

Joaquim Mullol reports personal fees and other from SANOFI‐GENZYME & REGENERON, personal fees and other from NOVARTIS, grants, personal fees and other from VIATRIS/MEDA Pharma, grants and personal fees from NOUCOR/URIACH Group, personal fees from Menarini, personal fees from UCB, personal fees and other from AstraZeneca, grants, personal fees and other from GSK, personal fees from MSD, personal fees and other from Lilly, personal fees and other from GLENMARK, outside the submitted work.

Alvaro A. Cruz reports personal fees from Astrazeneca, personal fees from Boehringer Ingelheim, personal fees from Chiesi, personal fees from GSK, personal fees from Eurofarma, personal fees from Myralis, personal fees from Farmoquimica, personal fees from Sanofi, personal fees from Abdi Ibrahim, personal fees from Novartis, outside the submitted work.

Juan Carlos Ivancevich reports personal fees from Laboratorios Casasco Argentina, outside the submitted work.

Sanna K. Toppila‐Salmi reports grants and personal fees from Sanofi Pharma, grants and personal fees from GSK, personal fees from AstraZeneca, personal fees from OrionPharma, personal fees from ALK‐Abelló, personal fees from Clario, outside the submitted work.

Joaquin Sastre reports grants and personal fees from SANOFI, personal fees from GSK, personal fees from NOVARTIS, personal fees from ASTRA ZENECA, personal fees from MUNDIPHARMA, personal fees from FAES FARMA, outside the submitted work.

Philippe Devillier reports personal fees and non‐financial support from Astra Zeneca, personal fees from Chiesi, personal fees from GlaxoSmithKline, personal fees and non‐financial support from ALk‐Abello, personal fees and non‐financial support from Stallergenes, personal fees from Menarini, personal fees from Viatris, personal fees from Procter & Gamble Heath, outside the submitted work.

Jonathan A. Bernstein reports personal fees from Pfizer, personal fees from Lilly, personal fees from Sanofi, outside the submitted work.

Désirée E. Larenas‐Linnemann reports personal fees from ALK, Astrazeneca national and global, Bayer, Chiesi, Grunenthal, Grin, GSK national and global, Viatris, Menarini, MSD, Novartis, Pfizer, Sanofi, Siegfried, Carnot, Syneos Health, grants from Abbvie, Bayer, Lilly, Sanofi, Astrazeneca, Pfizer, Novartis, Pulmonair, GSK, Chiesi, outside the submitted work; and Editor in chief of Immune System (Karger)Ðember of asthma committee ACAAIÐubgroup chair of allergen immunotherapy Practice parameter update JTF AAAAI/ACAAI 2024Ðember of allergen immunotherapy committee AAAAIÜhair of allergen immunotherapy committee CMICAÐember of allergic asthma task force EAACI.

Lucas Leemann reports grants from EU Horizon Grant, during the conduct of the study.

Torsten Zuberbier reports personal fees from Amgen, personal fees from AstraZeneca, personal fees from AbbVie, personal fees from ALK‐Abelló, personal fees from Almirall, personal fees from Astellas, personal fees from Bayer Health Care, personal fees from Bencard, personal fees from Berlin Chemie, personal fees from FAES Farma, personal fees from HAL Allergie GmbH, personal fees from Henkel, personal fees from Kryolan, personal fees from Leti, personal fees from L'Oreal, personal fees from Meda, personal fees from Menarini, personal fees from Merck Sharp & Dohme, personal fees from Novartis, personal fees from Nuocor, personal fees from Pfizer, personal fees from Sanofi, personal fees from Stallergenes, personal fees from Takeda, personal fees from Teva, personal fees from UCB, personal fees from Uriach, personal fees from Abivax, personal fees from Blueprint, personal fees from Celldex, personal fees from Celltrion, outside the submitted work; and Committee member, ‘Allergic Rhinitis and its Impact on Asthma’ (ARIA), Member of the Board, German Society for Allergy and Clinical Immunology (DGAKI), Head, European Centre for Allergy Research Foundation (ECARF), President, Global Allergy and Asthma Excellence Network (GA^2^LEN), and Member, Committee on Allergy Diagnosis and Molecular Allergology, World Allergy Organization (WAO).

Oscar Palomares reports fees for Lectures/Advisory Boards from AstraZeneca, Pfizer, GSK, Inmunotek S.L, Novartis, Sanofi‐Genezyme, and Regeneron. Oscar Palomares has received Research Grants from: MINECO, MICINN, CAM, Inmunotek S.L, Novartis and AstraZeneca.

Tomohisa Iinuma reports grants from Sanofi, outside the submitted work.

Pedro Carreiro‐Martins reports MSD (Grant for research project outside of this work).

Helga Kraxner reports Speaker's fee and congress support from SanofiÐpeaker's fee and congress support from ViatrisÐpeaker's fee and congress support from Berlin‐ChemieÐpeaker's fee and congress support from EwopharmaÚdvisory Board membership: Sanofi Údvisory Board membership: Berlin‐Chemie.

Brigita Gradauskiene reports grants and personal fees from AstraZeneca, personal fees from Berlin‐Chemie Menarini, Takeda, Viatris, AbbVie, outside the submitted work.

Piotr Kuna reports personal fees from Adamed, personal fees from Berlin Chemie Menarini, personal fees from AstraZeneca, personal fees from FAES, personal fees from Glenmark, personal fees from GSK, personal fees from Celon Pharma, personal fees from Novartis, personal fees from Polpharma, personal fees from Sandoz, personal fees from Sanofi, personal fees from Teva, outside the submitted work.

Michael Makris reports personal fees from SANOFI AVENTIS, personal fees from PFIZER, personal fees from ELPEN, personal fees from ASTRA ZENECA, personal fees from GSK, personal fees from NOVARTIS, outside the submitted work.

Luis Taborda‐Barata reports personal fees from LETI, personal fees from Novartis, outside the submitted work.

Charlotte Suppli Ulrik reports personal fees from AZ, personal fees from GSK, grants and personal fees from BI, personal fees from TEVA, personal fees from PFIZER, personal fees from ORION, grants and personal fees from SANOFI, personal fees and non‐financial support from NOVARTIS, personal fees from CHIESI, personal fees from COVIS PHARMA, personal fees from BERLIN CHEMIE, outside the submitted work.

Nicolas Roche reports grants and personal fees from Boehringer Ingelheim, grants and personal fees from Novartis, grants and personal fees from GSK, personal fees from AstraZeneca, personal fees from Chiesi, grants and personal fees from Pfizer, personal fees from Sanofi, personal fees from Zambon, personal fees from MSD, personal fees from Austral, personal fees from Biosency, outside the submitted work.

Heidi Olze received fees (lectures, advisory boards, research grants) from F. Hoffmann‐La Roche Ltd, Sanofi‐Aventis Deutschland GmbH, AstraZeneca GmbH, GlaxoSmithKline GmbH & Co. KG and Novartis, outside of the present work.

Jaime Correia‐de‐Sousa reports other from Boheringer Ingelheim, personal fees and other from GSK, grants, personal fees and other from AstraZeneca, from Bial, non‐financial support from Mundipharma, personal fees from Sanofi, other from Novartis, personal fees from MSD, personal fees from Medinfar, outside the submitted work.

Yoshitaka Okamoto reports personal fees from Torii pharmaceutical Co. Ltd, personal fees from Tanabe‐Mitsubishi Pharmaceutical Co. Ltd., personal fees from Kirin Holdings Co. Ltd., personal fees from Shionogi Co. Ltd., personal fees from Stallergenes‐Greer, personal fees from Diichi‐Sankyo, outside the submitted work.

Frederico S. Regateiro reports personal fees from Novartis, personal fees from Sanofi, personal fees from AstraZeneca, personal fees from GSK, personal fees from Medinfar, personal fees from Azentis, outside the submitted work.

Markus Ollert reports personal fees from Hycor Diagnostics, personal fees from Allergy Therapeutics, grants from Angany SA, outside the submitted work; and Scientific co‐founder of Tolerogenics SARL, Esch‐sur‐Alzette, Luxembourg.

Boleslaw Samolinski reports personal fees from Polpharma, personal fees from Viatris, grants and personal fees from AstraZeneca, personal fees from TEVA, personal fees from patient ombudsman, personal fees from Polish Allergology Society, grants from GSK, personal fees from ADAMED, outside the submitted work.

Ana Todo‐Bom reports personal fees from Leti, personal fees from GSK, personal fees from Mylan, grants from Abbvie, grants from Roxal, grants from Sanofi, outside the submitted work.

Marysia Recto reports honoraria from A. Menarini, Viatris, Glenmark, Bayer, Cathay Drug, outside the submitted work.

Margitta Worm reports other from Novartis Pharma GmbH, other from Parexel International, other from Sanofi‐Aventis Deutschland GmbH, other from Aimmune Therapeutics UK Limited, other from Bencard Allergie GmbH, other from Allergopharma GmbH & Co. KG, other from ALK‐Abelló Arzneimittel GmbH, other from Mylan Germany GmbH/Mice Service GmbH/Viatris, other from Leo Pharma GmbH, other from Almirall Hermal GmbH, other from Novartis AG, other from GlaxoSmithKline GmbH & Co. KG., other from AbbVie Deutschland GmbH & Co. KG, other from Lilly Deutschland GmbH, other from AstraZeneca GmbH, other from Pfizer Pharma GmbH, other from Boehringer Ingelheim Pharma GmbH & Co.KG, other from Amgen GmbH, other from FomF GmbH, outside the submitted work.

Lorenzo Cecchi reports personal fees from Thermofisher, personal fees from Novartis, personal fees from GSK, personal fees from Menarini, personal fees from Sanofi, personal fees from Astra Zeneca, personal fees from Chiesi, outside the submitted work.

Ioanna Tsiligianni reports grants from Chiesi, GSK Hellas, Menarini, Astra Zeneca Greece, outside the submitted work.

Holger J. Schünemann reports developed guidelines on Allergic Rhinitis in Asthma (ARIA) and his academic institution received research funding for it.

Sian Williams reports grants from ALK, outside the submitted work.

Mohamed H. Shamji reports grant from Immune Tolerance Network, grants from Medical Research Council, grants and personal fees from Allergy Therapeutics, grants and personal fees from LETI Laboratorios, grants from Rovolo Biotherapeutics, outside the submitted work.

The other authors have nothing to declare, outside the submitted paper.

## Disclaimer

Dr. Alkis Togias' co‐authorship of this publication does not constitute concurrence by the National Institute of Allergy and Infectious Diseases, the National Institutes of Health or any other agency of the United States Government.

## Supporting information


Supporting Information S1

## Data Availability

The data that support the findings of this study are available from the corresponding author upon reasonable request.
