# Supplementary material for: ARIA‐EAACI 2025: Person‐Centred, Digitally Enabled and Artificial Intelligence‐Assisted Change Management in Airway Diseases: An OECD Best Practice for Integrated Care for Chronic Diseases
Source: Clin Transl Allergy. 2026 Aug 18;16(8):e70192. doi: 10.1002/clt2.70192 (PMC13486308; doi:10.1002/clt2.70192)
Supplement: Supplementary file 1 [file CLT2-16-e70192-s001.docx]

**Table 1 online: Maturity level of MASK-air®** (adapted from ^1^)

| **Rhinitis platform** | **TRL** |  | **Asthma platform** | **TRL** |  |
| --- | --- | --- | --- | --- | --- |
| App for rhinitis and multimorbidity (MASK-air^®^): 28 countries, 17 languages | 9 | ^2-4^ | Adaptation of the MASK-air app for SA tested by the DHE SA-TWINNING | 8 | ^5^ |
| PROMs for rhinitis | 9 |  | PROMS for asthma | 9 | ^6^ |
| CARAT questionnaire for screening and control of rhinitis and asthma, 20 countries | 9 | ^7-10^ | The same questionnaire will be used | 9 | ^7-9^ |
| e-physician questionnaire for rhinitis (available on the MASK-air website) deployed in 28 countries and 20 languages | 9 | ^11^ | Adaptation of the MASK questionnaire for SA developed by the DHE SA-TWINNING | 6 | ^5,12^ |
| Electronic CDSS in English for rhinitis | 8 | ^13^ |  |  |  |
| Embedding air quality (outdoor air pollution) and pollen data in MASK-air^®^ (POLLAR) | 9 | ^14^ | Alerts for air pollution and pollens predicting asthma exacerbations | 5 |  |
|  |  |  | Alerts for rhinovirus predicting asthma exacerbations | 4 | ^15,16^ |
| EAACI-ARIACARE-digital network (28 countries, 20 languages) | 9 |  | The same network will be used | 9 |  |
| Symptom-medication score for rhinitis | 9 | ^17^ | Daily control-medication score for asthma | 5 |  |
|  | NA |  | Sensors for pulmonary function | 3 |  |
| Embedding artificial intelligence in MASK-air^®^ | 4 |  | Embedding artificial intelligence | 4 |  |

**Table 2 online: Methodologic validation of MASK-air®** (updated from ^18^)

|  | **Study name** | **Ref** | **Study type** | **N users** | **N days** | **N countries** |
| --- | --- | --- | --- | --- | --- | --- |
| **Methodology** | COSMIN guidelines | ^19^ | Obs, CS-L | 2,497 | 14,612* | 15 |
|  | Test-retest, intra-class coefficient | ^20^ | Obs, CS-L | 17,780 | 317,176 | 25 |
|  | Quality of data (intra-individual response variability) | ^17^ | Obs, CS | 14,189 | 205,904 | 23 |
|  | Independence of data | ^20^ | Obs, CS | 1,136 | 5,889 | 18 |
|  | VAS work | ^17,21-23^ | Obs, CS | 14,189 | 205,904 | 18 |
|  | EQ-5D VAS | ^22,23^ | Obs, CS | 1,288 | NA | 18 |
| **Impact** | EQ-5D domains | ^21-24^ | Obs, CS |  |  | 26 |
|  | Work Productivity and Activity Impairment Allergic Specific (WPAI:AS) | ^21-24^ | Obs, CS | 1,288 | NA | 18 |
|  | CARAT | ^10^ | Obs, CS | 1,086 | 2,042 | 22 |
|  | *CARAT** | *^25^* | *Systematic review* |  |  |  |
| **PROMs** | Validation of PROMs | ^26^ |  |  |  |  |
|  | PROMs in adolescents and young adults | ^27^ | Obs, CS | | | |
|  | PROMs in severe asthma |  | Obs, CS | 86 | 2,349 | 12 |
|  | Asthma and small airways | ^28^ | Obs, CS |  |  | Turkey |
|  | MASK-air in old age adults | ^29^ | Obs, CS | 19,888 | 349,045 | 27 |
|  | Combined symptom-medication scores for allergic rhinitis (ARIA-EAACI CSMS) | ^30^ | Obs, CS | 17,780 | 317,176 | 25 |
|  | Electronic daily control-medication score in asthma (e-DASTHMA) | ^31^ | Obs, CS | 1662 | 135,635 | 25 |
|  | Cut-off levels | ^32^ | Obs, CS-L | 23,201 | 395,223 | 27 |
|  | Mobile health app for monitoring AR and asthma in real life in Lithuanian MASK-air users | ^33^ | Obs, CS-L | 149 | NA | Lithuania |
|  | Implementation of the MASK-air^®^ app for rhinitis and asthma in old age adults. MASK@Puglia | ^34^ | Obs, CS | 174 | NA | Italy |
| **Patient’s perception** | Impact of MASK-air on patients | ^35^ | Obs, CS | 131 | NA | Poland |
|  | Factors Affecting Usage of a MASK-air® by Old-Age Asthmatics Living in Inner Central Portugal | ^36^ | Obs, CS | 72 |  | Portugal |
|  | Comparison of anti-histamine reporting by MASK-air, Google Trends and sales in Europe | ^37^ | Obs, CS |  |  |  |
|  | CDSS (Clinical Decision Support System) | ^38^ |  | NA | NA | NA |
|  | CHRODIS guidelines | ^39^ |  | NA | NA | NA |
|  | Patient’s satisfaction | ^40,41^ | Obs, CS | 1691 | 28,177 | Europe |
| **Privacy** | Geolocation (GDPR) | ^42^ | Obs, CS | 8535 | 93,116 | 22 |
|  | GDPR | ^43^ |  | NA | NA | NA |

ARIA: Allergic Rhinitis and its Impact on Asthma, CARAT: Control of Rhinitis and Asthma test, CHRODIS: Joint Action on Chronic Diseases, COSMIN: COnsensus-based Standards for the selection of health Measurement INstruments, EAACI: European Academy of Allergy and Clinical Immunology, EQ-5D: EuroQol, GDPR: General Data Protection Regulation, PROMs: Patient-reported outcome measure, VAS: visual analogue scale. Obs: Observational study, CS: Cross-sectional study, L: Longitudinal study, NA: not applicable*: Systematic review carried out for MASK-air

**Table 3 online: Strategic overview** (adapted from ^1^)

|  | **Acronym & ref** | **Name** | **Dates** |
| --- | --- | --- | --- |
| **WHO-associated projects** | | | |
|  | GARD ^44^ | Global Alliance against Chronic Respiratory Diseases, demonstration project | 2003-23 |
|  | WHO-ITU ^45^ | “Be He@lthy, Be Mobile” handbook on asthma and COPD | 2017 |
| **EU grants and projects** | | | |
|  | EIP on AHA ^46^ | European Innovation Partnership on Active and Healthy Ageing (DG Santé & CONNECT) | 2012-20 |
|  | Joint Research Center (JRC) Scientific and Policy Reports on Strategic Intelligence Monitor on Personal Health Systems Phase 3 (SIMPHS3) ^47^ | | 2015 |
|  | MACVIA ^23^ | European Regional Development Fund (ERDF-Région Languedoc-Roussillon) | 2016-7 |
|  | Twinning ^11^ | Transfer of Innovation (DG Santé & CONNECT) | 2017-9 |
|  | DHE Twinning ^5^ | Transfer of innovation in severe asthma (H2020) | 2019-20 |
|  | POLLAR ^12,14^ | Impact of air Pollution on Asthma and Rhinitis (EIT Health) | 2018-9 |
|  | CATALYSE ^48^ | Climate change (Horizon Europe) | 2022-6 |
|  | MASK@PACA | European Regional Development Fund (ERDF-Région PACA) | 2021-2 |
|  | CAMBAS | Horizon Europe | 2026- |
|  | ClimAir | Horizon Europe | 2026- |
|  | Good Practice DG Santé on digital health (DG Santé) ^49^ | | 2018 |
|  | Best Practice OECD-DG Santé ^50,51^ | | 2023 |
|  | Accreditation by Polish Government | | 2024-6 |
| **ARIA-EAACI Task Forces and Position Papers** | | | |
|  | Electronic daily symptom-control score in allergy (CSMS) ^30,31,52^ | | 2022 |
|  | Electronic daily symptom-control score in asthma (e-DASTHMA) ^31,52^ | | 2023 |
|  | Digital biomarkers in asthma ^53^ | | 2024 |
|  | Digital biomarkers in allergen immunotherapy ^54^ | | 2024 |
|  | UCRAID ^55^ | | 2023 |

ARIA: Allergic Rhinitis and its Impact on Asthma, CARAT: Control of Allergic Rhinitis and Asthma Test, EAACI: European Academy of Allergy and Clinical Immunology, e-CDSS: electronic clinical decision support system, GA^2^LEN: Global Allergy and Asthma European Network, GARD: Global Alliance against Chronic Respiratory Diseases, OECD: Organisation for Economic Co-operation and Development, POLLAR: Impact of air Pollution on Asthma and Rhinitis, CATALYSE: Climate Action to Advance HeaLthY Societies in Europe, WHO: World Health Organization. ITU: International Telecommunication Union, UCRAID/ Ukrainian Citizen and refugee electronic support in Respiratory diseases, Allergy, Immunology and Dermatology

**References**

1.	Bousquet J, Anto JM, Bachert C, et al. ARIA digital anamorphosis: Digital transformation of health and care in airway diseases from research to practice. *Allergy*. Jan 2021;76(1):168–190. doi:10.1111/all.14422

2.	Bousquet J, Arnavielhe S, Bedbrook A, et al. MASK 2017: ARIA digitally-enabled, integrated, person-centred care for rhinitis and asthma multimorbidity using real-world-evidence. *Clin Transl Allergy*. 2018;8:45. doi:10.1186/s13601-018-0227-6

3.	Bousquet J, Devillier P, Anto JM, et al. Daily allergic multimorbidity in rhinitis using mobile technology: A novel concept of the MASK study. *Allergy*. Aug 2018;73(8):1622–1631. doi:10.1111/all.13448

4.	Bedard A, Basagana X, Anto JM, et al. Mobile technology offers novel insights into the control and treatment of allergic rhinitis: The MASK study. *J Allergy Clin Immunol*. Jul 2019;144(1):135–143 e6. doi:10.1016/j.jaci.2019.01.053

5.	Bousquet J, Bedbrook A, Czarlewski W, et al. Digital Health Europe (DHE) Twinning on severe asthma-kick-off meeting report. *J Thorac Dis*. May 2021;13(5):3215–3225. doi:10.21037/jtd-21-792

6.	Sousa-Pinto B, Fonseca JA, Gemicioglu B, et al. Patient-reported outcome measures (PROMs) using the MASK-air(R) app in severe asthma. *Allergy*. Feb 8 2022;77(5):1600–1602. doi:10.1111/all.15248

7.	Azevedo P, Correia de Sousa J, Bousquet J, et al. Control of Allergic Rhinitis and Asthma Test (CARAT): dissemination and applications in primary care. *Prim Care Respir J*. Mar 2013;22(1):112–6. doi:10.4104/pcrj.2013.00012

8.	Fonseca JA, Nogueira-Silva L, Morais-Almeida M, et al. Validation of a questionnaire (CARAT10) to assess rhinitis and asthma in patients with asthma. *Allergy*. Aug 2010;65(8):1042–8. doi:ALL2310 [pii]

10.1111/j.1398-9995.2009.02310.x

9.	van der Leeuw S, van der Molen T, Dekhuijzen PN, et al. The minimal clinically important difference of the control of allergic rhinitis and asthma test (CARAT): cross-cultural validation and relation with pollen counts. *NPJ Prim Care Respir Med*. 2015;25:14107. doi:10.1038/npjpcrm.2014.107

10.	Sousa-Pinto B, Sa-Sousa A, Amaral R, et al. Assessment of the Control of Allergic Rhinitis and Asthma Test (CARAT) using MASK-air. *J Allergy Clin Immunol Pract*. Jan 2022;10(1):343–345 e2. doi:10.1016/j.jaip.2021.09.012

11.	Bousquet J, Agache I, Aliberti MR, et al. Transfer of innovation on allergic rhinitis and asthma multimorbidity in the elderly (MACVIA-ARIA) - Reference Site Twinning (EIP on AHA). *Allergy*. Jun 10 2017;73(1):77–92. doi:10.1111/all.13218

12.	Sofiev M, Palamarchuk Y, Bedard A, et al. A demonstration project of Global Alliance against Chronic Respiratory Diseases: Prediction of interactions between air pollution and allergen exposure-the Mobile Airways Sentinel NetworK-Impact of air POLLution on Asthma and Rhinitis approach. *Chin Med J (Engl)*. Jul 5 2020;133(13):1561–1567. doi:10.1097/CM9.0000000000000916

13.	Courbis AL, Murray RB, Arnavielhe S, et al. Electronic Clinical Decision Support System for allergic rhinitis management: MASK e-CDSS. *Clin Exp Allergy*. Dec 2018;48(12):1640–1653. doi:10.1111/cea.13230

14.	Bousquet J, Anto JM, Annesi-Maesano I, et al. POLLAR: Impact of air POLLution on Asthma and Rhinitis; a European Institute of Innovation and Technology Health (EIT Health) project. *Clin Transl Allergy*. 2018;8:36. doi:10.1186/s13601-018-0221-z

15.	Sousa-Pinto B, Anto JM, Sheikh A, et al. Comparison of epidemiologic surveillance and Google Trends data on asthma and allergic rhinitis in England. *Allergy*. Feb 2022;77(2):675–678. doi:10.1111/all.15139

16.	Sousa-Pinto B, Halonen JI, Anto A, et al. Prediction of Asthma Hospitalizations for the Common Cold Using Google Trends: Infodemiology Study. *J Med Internet Res*. Jul 6 2021;23(7):e27044. doi:10.2196/27044

17.	Bedard A, Anto JM, Fonseca JA, et al. Correlation between work impairment, scores of rhinitis severity and asthma using the MASK-air((R)) App. *Allergy*. Jul 2020;75(7):1672–1688. doi:10.1111/all.14204

18.	Bousquet J, Anto JM, Sousa-Pinto B, et al. Digitally-enabled, patient-centred care in rhinitis and asthma multimorbidity: The ARIA-MASK-air((R)) approach. *Clin Transl Allergy*. Jan 2023;13(1):e12215. doi:10.1002/clt2.12215

19.	Caimmi D, Baiz N, Tanno LK, et al. Validation of the MASK-rhinitis visual analogue scale on smartphone screens to assess allergic rhinitis control. *Clin Exp Allergy*. Dec 2017;47(12):1526–1533. doi:10.1111/cea.13025

20.	Sousa-Pinto B, Eklund P, Pfaar O, et al. Validity, reliability, and responsiveness of daily monitoring visual analog scales in MASK-air(R). *Clin Transl Allergy*. Aug 2021;11(7):e12062. doi:10.1002/clt2.12062

21.	Bousquet J, Bewick M, Arnavielhe S, et al. Work productivity in rhinitis using cell phones: The MASK pilot study. *Allergy*. Oct 2017;72(10):1475–1484. doi:10.1111/all.13177

22.	Bousquet J, VandenPlas O, Bewick M, et al. The Work Productivity and Activity Impairment Allergic Specific (WPAI-AS) Questionnaire Using Mobile Technology: The MASK Study. *J Investig Allergol Clin Immunol*. 2018;28(1):42–44. doi:10.18176/jiaci.0197

23.	Bousquet J, Arnavielhe S, Bedbrook A, et al. The Allergic Rhinitis and its Impact on Asthma (ARIA) score of allergic rhinitis using mobile technology correlates with quality of life: The MASK study. *Allergy*. Feb 2018;73(2):505–510. doi:10.1111/all.13307

24.	Vandenplas O, Suarthana E, Rifflart C, Lemiere C, Le Moual N, Bousquet J. The Impact of Work-Related Rhinitis on Quality of Life and Work Productivity: A General Workforce-Based Survey. *J Allergy Clin Immunol Pract*. Jan 28 2020;doi:10.1016/j.jaip.2019.12.033

25.	Vieira RJ, Sousa-Pinto B, Cardoso-Fernandes A, et al. Control of Allergic Rhinitis and Asthma Test: A systematic review of measurement properties and COSMIN analysis. *Clin Transl Allergy*. Sep 2022;12(9):e12194. doi:10.1002/clt2.12194

26.	Bousquet J, Sousa-Pinto B, Anto JM, et al. Concurrent validity, cut-offs and ability to change of patient-reported outcome measures for rhinitis and asthma in MASK-air((R)). *Clin Transl Allergy*. Sep 2024;14(9):e12390. doi:10.1002/clt2.12390

27.	Sousa-Pinto B, Valiulis A, Melen E, et al. Asthma and rhinitis control in adolescents and young adults: A real-world MASK-air study. *Pediatr Allergy Immunol*. Feb 2024;35(2):e14080. doi:10.1111/pai.14080

28.	Vardaloglu I, Sousa-Pinto B, Bousquet J, et al. In symptomatic patients on as-needed inhaled corticosteroids-formoterol, VAS asthma is associated with small airways resistance. *J Asthma*. Feb 2024;61(2):132–139. doi:10.1080/02770903.2023.2248485

29.	Taborda-Barata L, Ventura M, Blain H, et al. MASK-air® real-world data in respiratory allergy in old-age adults. *Clin Transl Allergy*. 2022;

30.	Sousa-Pinto B, Azevedo LF, Jutel M, et al. Development and validation of combined symptom-medication scores for allergic rhinitis. *Allergy*. Dec 21 2022;77(7):2147–2162. doi:10.1111/all.15199

31.	Sousa-Pinto B, Jacome C, Perreira A, et al. Development and validation of an electronic daily control score for asthma (e-DASTHMA). *Lancet Digital Health*. 2023;

32.	Sousa-Pinto B, Sa-Sousa A, Vieira RJ, et al. Cut-off values of MASK-air(R) Patient-Reported Outcome Measures (PROMs). *J Allergy Clin Immunol Pract*. Dec 22 2022;doi:10.1016/j.jaip.2022.12.005

33.	Kvedariene V, Biliute G, Didziokaite G, et al. Mobile health app for monitoring allergic rhinitis and asthma in real life in Lithuanian MASK-air users. *Clin Transl Allergy*. Sep 2022;12(9):e12192. doi:10.1002/clt2.12192

34.	Ventura MT, Giuliano AFM, Buquicchio R, et al. Implementation of the MASK-Air(R) App for Rhinitis and Asthma in Older Adults: MASK@Puglia Pilot Study. *Int Arch Allergy Immunol*. 2022;183(1):45–50. doi:10.1159/000518032

35.	Szylling A, Samolinski B, Raciborski F, et al. Factors that influence user adherence of the Mask-air(R) application. *Clin Transl Allergy*. Apr 2025;15(4):e70054. doi:10.1002/clt2.70054

36.	Abreu MIT, Santos AF, Gama JMR, et al. Factors Affecting Usage of a Digital Asthma Monitoring Application by Old-Age Asthmatics Living in Inner Central Portugal. *Clin Interv Aging*. 2024;19:971–979. doi:10.2147/CIA.S448797

37.	Vieira R, Sousa-Pinto B, Anto J, et al. Usage patterns of oral H1-antihistamines in 10 European countries: A study using MASK-air® and Google Trends real-world data. *World Allergy Organ J*. 2022;15:100660.

38.	Courbis AL, Murray RB, Arnavielhe S, et al. Electronic Clinical Decision Support System for allergic rhinitis management: MASK e-CDSS. *Clin Exp Allergy*. Jul 12 2018;48(12):1640–53. doi:10.1111/cea.13230

39.	Bousquet J, Onorato GL, Bachert C, et al. CHRODIS criteria applied to the MASK (MACVIA-ARIA Sentinel NetworK) Good Practice in allergic rhinitis: a SUNFRAIL report. *Clin Transl Allergy*. 2017;7:37. doi:10.1186/s13601-017-0173-8

40.	Sousa-Pinto B, Vieira RJ, Bognanni A, et al. Comparison of Allergic Rhinitis Treatments on Patient Satisfaction: A MASK-air and EAACI Methodological Committee Report. *Allergy*. Sep 26 2025;doi:10.1111/all.70055

41.	Vieira RJ, Sousa-Pinto B, Gil-Mata S, et al. Comparing allergic rhinitis treatments based on patient satisfaction: A MASK-air(R) study. *J Allergy Clin Immunol Pract*. Jun 6 2025;doi:10.1016/j.jaip.2025.05.058

42.	Samreth D, Arnavielhe S, Ingenrieth F, et al. Geolocation with respect to personal privacy for the Allergy Diary app - a MASK study. *World Allergy Organ J*. 2018;11(1):15. doi:10.1186/s40413-018-0194-3

43.	Laune D, Arnavielhe S, Viart F, et al. [Adaptation of the General Data Protection Regulation (GDPR) to a smartphone app for rhinitis and asthma (MASK-air(R))]. *Rev Mal Respir*. Nov 2019;36(9):1019–1031. Reglement general sur la protection des donnees pour MASK-air(R) (application mobile rhinite et asthme). doi:10.1016/j.rmr.2019.08.003

44.	Bousquet J, Dahl R, Khaltaev N. Global alliance against chronic respiratory diseases. *Allergy*. Mar 2007;62(3):216–23. doi:10.1111/j.1398-9995.2007.01307.x

45.	Mobile Health for Asthma and Chronic Obstructive Respiratory Disease (mBreatheFreely). World Health Organisation and International Telecommunication Union. [*https://wwwwhoint/publications/i/item/9789241514002*](https://wwwwhoint/publications/i/item/9789241514002). 2017;

46.	Bousquet J, Illario M, Farrell J, et al. The Reference Site Collaborative Network of the European Innovation Partnership on Active and Healthy Ageing. *Transl Med UniSa*. Jan–Jun 2019;19:66–81.

47.	Bousquet J, Mercier J, Avignon A, Camuzat T, Abadie F. Strategic Intelligence Monitor on Personal Health Systems Phase 3 (SIMPHS3)

MACVIA-LR (France) Case Study Report. Science for Policy. [*https://publicationsjrceceuropaeu/repository/handle/JRC94487?mode=full*](https://publicationsjrceceuropaeu/repository/handle/JRC94487?mode=full). 2015;

48.	Sousa-Pinto B, Palamarchuk Y, Leemann L, et al. From MASK-air and SILAM to CATALYSE (Climate Action To Advance HeaLthY Societies in Europe). *J Investig Allergol Clin Immunol*. Feb 22 2024;34(1):12–19. doi:10.18176/jiaci.0923

49.	Bousquet J, Bedbrook A, Czarlewski W, et al. Guidance to 2018 good practice: ARIA digitally-enabled, integrated, person-centred care for rhinitis and asthma. *Clin Transl Allergy*. 2019;9:16. doi:10.1186/s13601-019-0252-0

50.	Integrating Care to Prevent and Manage Chronic Diseases : Best Practices in Public Health, OECD. *Éditions OCDE, Paris,* [*https://doiorg/101787/9acc1b1d-en*](https://doiorg/101787/9acc1b1d-en). 2023;

51.	Bousquet J, Sousa-Pinto B, Anto JM, et al. MASK-air(R): An OECD (Organisation for Economic Co-operation and Development) Best Practice for Public Health on integrated care for chronic diseases. *J Allergy Clin Immunol Pract*. Mar 21 2024;doi:10.1016/j.jaip.2024.03.024

52.	Bousquet J, Shamji MH, Anto JM, et al. Patient-centred digital biomarkers for allergic respiratory diseases and asthma: the ARIA-EAACI approach. *Allergy*. Apr 11 2023;doi:10.1111/all.15740

53.	Bousquet J, Shamji MH, Anto JM, et al. Patient-centered digital biomarkers for allergic respiratory diseases and asthma: The ARIA-EAACI approach - ARIA-EAACI Task Force Report. *Allergy*. Jul 2023;78(7):1758–1776. doi:10.1111/all.15740

54.	Pfaar O, Sousa-Pinto B, Papadopoulos NG, et al. Digitally-enabled, person-centred care (PCC) in allergen immunotherapy: An ARIA-EAACI Position Paper. *Allergy*. May 3 2024;doi:10.1111/all.16135

55.	Bousquet J, Samolinski B, Kaidashev I, et al. UCRAID (Ukrainian Citizen and refugee electronic support in Respiratory diseases, Allergy, Immunology and Dermatology) action plan. *Allergy*. Aug 28 2023;78(10):2581–2595. doi:10.1111/all.15855
